# Application of phase-change materials in memory taxonomy

**DOI:** 10.1080/14686996.2017.1332455

**Published:** 2017-06-13

**Authors:** Lei Wang, Liang Tu, Jing Wen

**Affiliations:** ^a^ School of Information Engineering, Nanchang HangKong University, Nanchang, P.R. China

**Keywords:** Phase-change materials, optical disc, scanning probe, random access, nanophotonic, memory taxonomy, 40 Optical, magnetic and electronic device materials, 201 Electronics / Semiconductor / TCOs

## Abstract

Phase-change materials are suitable for data storage because they exhibit reversible transitions between crystalline and amorphous states that have distinguishable electrical and optical properties. Consequently, these materials find applications in diverse memory devices ranging from conventional optical discs to emerging nanophotonic devices. Current research efforts are mostly devoted to phase-change random access memory, whereas the applications of phase-change materials in other types of memory devices are rarely reported. Here we review the physical principles of phase-change materials and devices aiming to help researchers understand the concept of phase-change memory. We classify phase-change memory devices into phase-change optical disc, phase-change scanning probe memory, phase-change random access memory, and phase-change nanophotonic device, according to their locations in memory hierarchy. For each device type we discuss the physical principles in conjunction with merits and weakness for data storage applications. We also outline state-of-the-art technologies and future prospects.

## Graphical abstract

**Figure:**
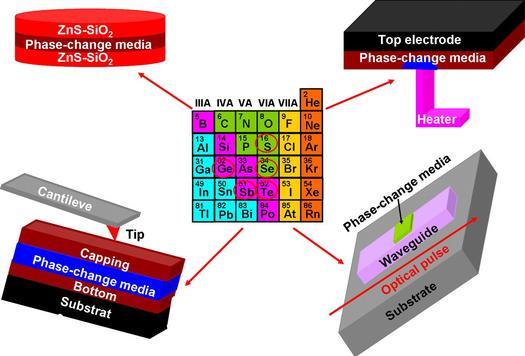

## 1. Introduction

**Figure 1.**
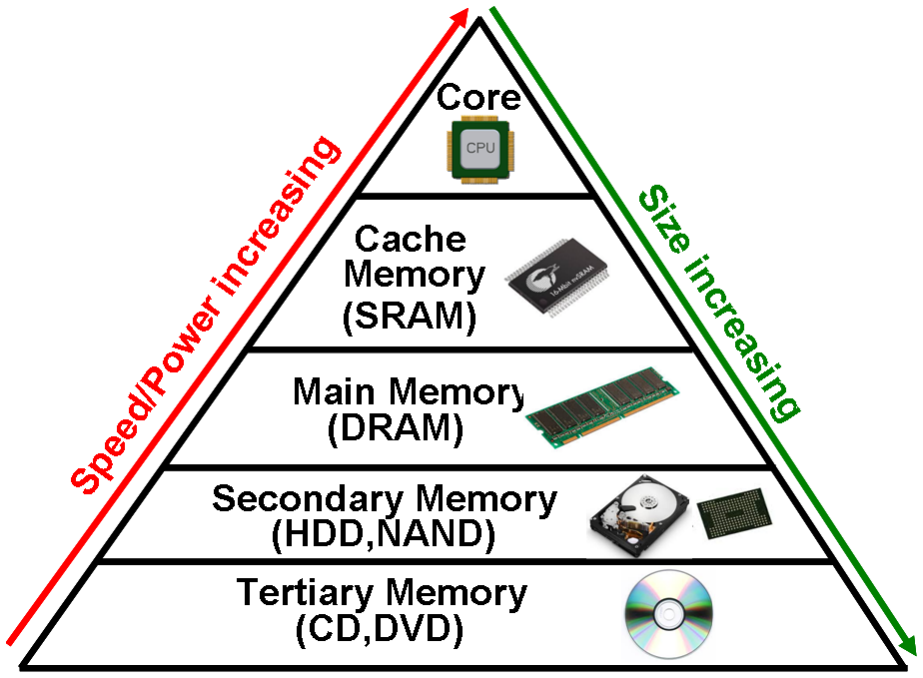
An example of memory taxonomy.

Today’s world is in an age of ‘information explosion’ where the total amount of digital data has been increasing at a phenomenal rate annually due to the rising popularity of mobile terminals, social networking services and business cloud services. This explosive growth obviously brings a crisis to conventional data storage devices, while creating a bright future for some emerging storage devices. A typical memory taxonomy, shown in Figure 1, consists of a processing core, cache memory, main memory, secondary memory, and tertiary memory from the top to the bottom of the pyramid. The processing core is used to execute arithmetic and logic calculations and various operations and therefore it has the fastest speed and the least data capacity when compared with other levels. The main function of cache memory is to store the most frequently used data in the main memory so as to reduce the time that the processing core takes to access the main memory. Consequently, cache memory is required to be high speed but with a small storage capacity [1]. For this reason, static random access memory (SRAM) that makes use of bistable latching circuitry (flip-flop) to store data is widely adopted for cache memory. The main memory stores the active programs and data that the processing core needs to read and execute, whereby it exhibits a fast speed and moderate capacity at an affordable price. A typical representative of main memory is volatile dynamic RAM (DRAM) that restores data by refreshing the capacitor [2]. Due to the relatively small capacity of DRAM, external devices with a larger storage capacity are preferable in order to store inactive programs and data, resulting in the advent of secondary memory devices such as hard disc drives (HDD) and flash negative-AND (NAND) memory. Accordingly, secondary memory has a much larger capacity than main memory in conjunction with a relatively fast speed. On the bottom of the memory pyramid is tertiary memory that is mainly employed to store rarely used archival data. Tertiary memory usually has a large capacity and a very slow access time and is exemplified by compact discs (CD) and digital versatile discs (DVD).

It should be noted, however, that such a memory hierarchy is no longer able to satisfy storage requirements for contemporary memory devices owing to the intensive development of information and communication technologies. Firstly, because of the column access strobe (CAS) latency, the main memory usually runs at a much slower speed than the processing core and cache memory. Such a speed gap also exists between the main memory and secondary memory. Secondly, the fact that the main memory needs stand-by power to retain data gives rise to extra energy consumption. Finally, the severe physical limits that the secondary/tertiary memory devices currently face (e.g. superparamagnetism limit for HDD [3], diffraction limit for DVD [4], and scaling limit for flash NAND [5]) have gradually eroded their potential to outpace the drastic increase of the archival data to date.

**Figure 2.**
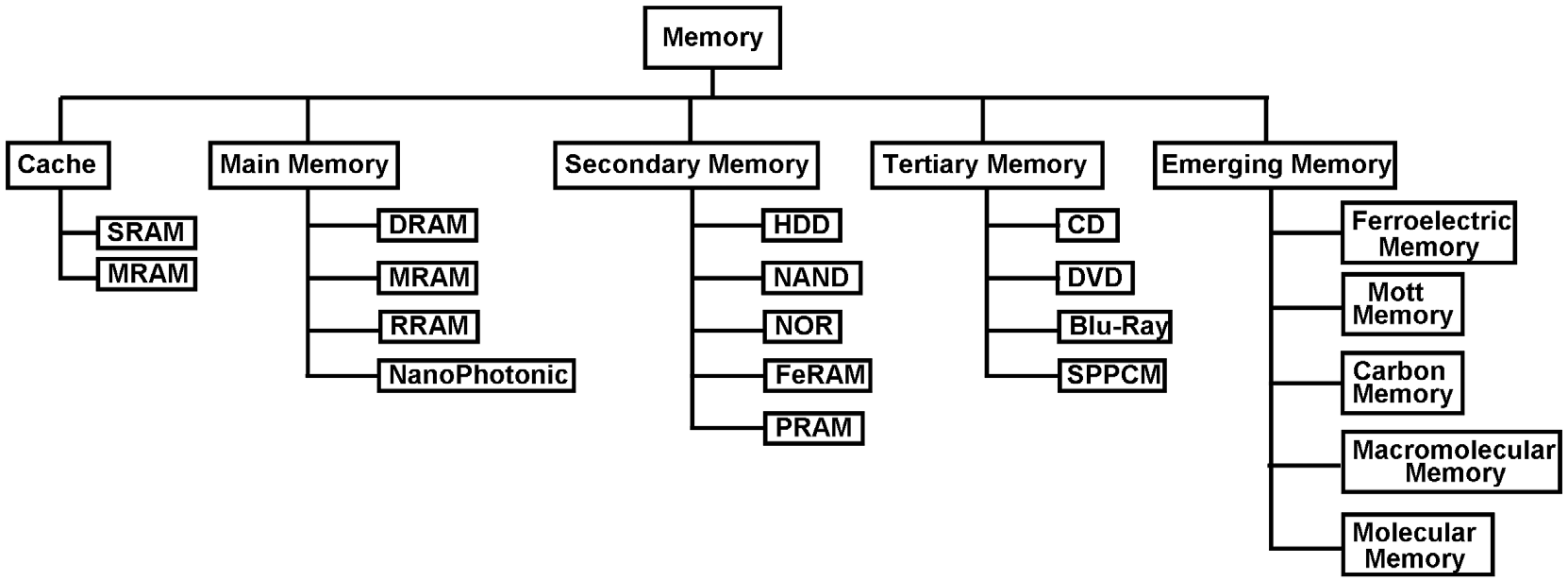
A modified memory taxonomy for the age of ‘big data’.

There is no doubt that the current world is in the age of ‘big data’ where everything is linked to a data source and every citizen’s daily life is captured digitally. Under these circumstances, the memory hierarchy shown in Figure 1 may not apply to the current situation and the necessity to develop and commercialize emerging memory devices for non-volatility, low power consumption, fast switching speed, and ultra-high storage density becomes increasingly important. Fortunately, triggered by booming material and electronic technologies, a large variety of emerging memory concepts have been proposed or even accomplished at the laboratory level during last two decades, to extend conventional storage memories beyond their fundamental scaling limits and thus to fulfil the storage demands of consumers worldwide. A modified memory taxonomy containing a combination of emerging memory devices with conventional devices is illustrated in Figure 2. A comparison of Figures 1 and 2 reveals several intriguing points. Note that the spiral of the pyramid in Figure 1 is not marked here as to date no alternatives have been found to even partially replace the silicon complementary metal-oxide-semiconductor (CMOS)-based core. In addition, some emerging memory devices have exhibited similar physical performances to their predecessors. For instance, magnetic RAM (MRAM) can exhibit a comparable endurance to SRAM [6,7], whereas the merits of DRAM, such as fast switching speed and long endurance cycles, can also be achieved by MRAM [6–8], resistive RAM (RRAM) [9,10], and a newly proposed nanophotonic device [11,12]. Meanwhile, the nanodevices that adopt phase-change materials (PCMs) as storage media have attained tremendous attention in the last decade, leading to the debut of phase-change RAM (PRAM) for secondary storage [13–16], and scanning probe phase-change memory (SPPCM) for tertiary storage [17–20]. However, although these newly developed devices can rival or even outperform their predecessors in some aspects of their physical performances, they are inevitably facing some serious physical limits in terms of the power consumption and scaling dimension. Therefore, most of the aforementioned devices, such as MRAM, RRAM, and PRAM, are still in the stage of prototype or early production, and lag far behind commercialization [21,22]. In order to overcome the physical drawbacks of these ‘prototypical’ memories and thus provide a technology to extend CMOS beyond its fundamental scaling limit, several non-volatile memory concepts that involves novel mechanism and materials different from the mature memories have been proposed, such as ferroelectric memory [23], mott memory [24], carbon memory [25], macromolecular memory [26], and molecular memory [27]. These emerging memories undoubtedly supplement the current cognitions on the physical principles of non-volatile memories, and provide potential solutions to the continued scaling of information processing technology. Nevertheless, these technologies are in a nascent stage where the corresponding physical properties remain unclear. It is not expected that they can compete with and replace the current mainstream storage devices in the nearest future. Hence, it would be sensible to spend more research on those ‘prototype’ established memories whose storage performances are yet to be fully explored than on either conventional memories with no further scaling potential or on the latest memories with unclear storage potential.

Based on the discussions presented so far, the ‘prototypical’ memory category is considered a more promising candidate to other categories, whereby more attention should be paid to its members that consist of MRAM, ferroelectric RAM (FeRAM), RRAM, and phase-change memories that can be sub-divided into PRAM, SPPCM, and nanophotonic memory using PCMs. As mentioned above, MRAM can be considered to be an appropriate replacement for SRAM in terms of its extremely high endurance cycles and ultra-fast speed. However, the ability of MRAM to maintain a sufficiently high electric field for storage purposes remains questionable when undergoing the continued downscaling process [28]. FeRAM is mainly implemented for embedded memory applications due to its low power consumption, long endurance, and fast speed [29], while its low data density has severely limited the prospect of FeRAM [30]. RRAM that functionalizes storage through a switching process between two distinct resistive states has recently gained much interest [9,31,32], as it primarily operates on the atomic level rather than storage charge level. As a consequence, the storage capacity of the RRAM strongly depends on the number of atoms that provide the electrical characteristics, while its storage mechanism is not yet fully understood. Therefore, the current consensus is that phase-change memories are more eligible to win the nomination to so-called ‘universal’ memory than its compatriots. First, the applications of phase-change memories cover a wide range of the memory hierarchy shown in Figure 2 from tertiary memory to main memory. Such excellent compatibility combined with recent findings that phase-change memories can perform some analogous arithmetic and logic computation to a CMOS-based processing core [33–36] has made the concept of a phase-change computer more viable. In addition, the inherent physical properties of the PCMs endow phase-change memory with several performance superiorities over other ‘prototypical’ memories. Most importantly, phase-change memories have been the subject of intensive research during the last two decades that brought a wealth of knowledge about their fundamental electrical, thermal, optical, and mechanical properties, in conjunction with the design of reliable device structures. Hence a comprehensive review of the physical principles of various phase-change memory devices, ranging from optical discs to phase-change nanophotonic devices and state-of-the-art phase-change memories, would help researchers understand and improve phase-change storage technologies. In this paper, the commonly used PCMs for storage memories are first introduced, followed by a detailed review of their respective applications for each memory hierarchy. Some new insights about the future development of phase-change memories are also presented.

## 2. Phase-change materials

**Figure 3.**
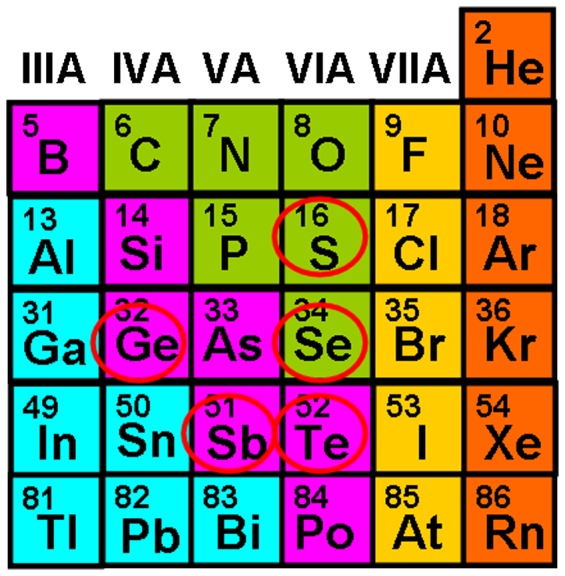
Typical elements in the chalcogenide family used for memory.

Materials for storage applications are usually required to have the following properties [37]: great scalability for ultra-high capacity; rapid switch among different phases for fast write/read speed; excellent stability at room temperature for long retention; and high contrast on certain physical properties among different phases. It is well known that PCMs can exist in both amorphous and crystalline states, while most of them rarely exhibit satisfactory biphasic stability and proper transition dynamic simultaneously for storage requirements. Moreover, the crystallization time commonly considered as the benchmark of the switching speed varies enormously for different PCMs and only those materials with a nanosecond switching regime become suitable for memory devices. Owing to these stringent requirements, the category of PCMs that can meet the aforementioned demands is mainly focused on the chalcogenide family (Group 16 elements, mainly S, Se, and Te) as well as their derivatives, as illustrated in Figure 3.

**Figure 4.**
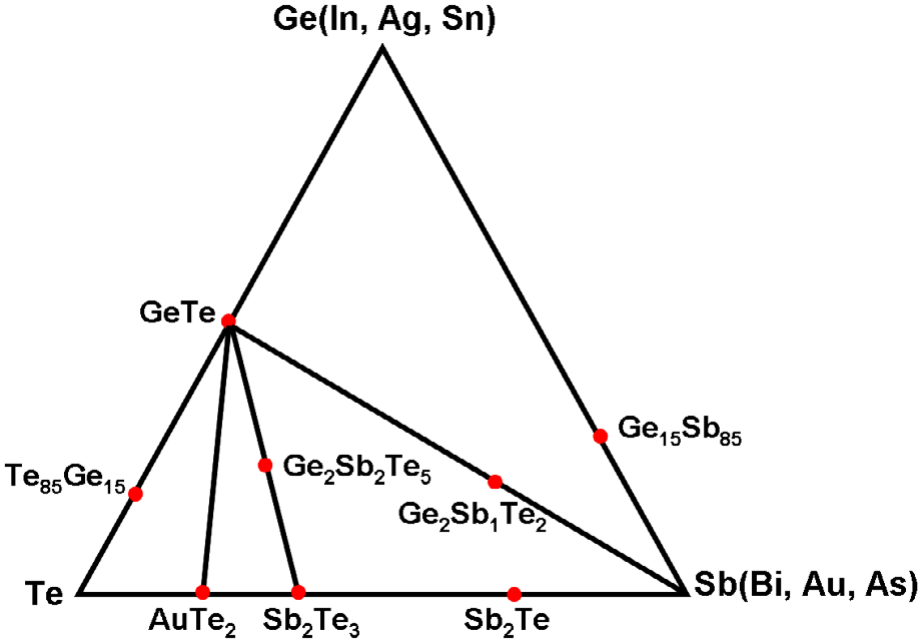
Tertiary Ge-Sb-Te phase diagram with some popular chalcogenide alloys highlighted.

Although the phenomenon that the electrical and optical properties of chalcogenide members can vary significantly between amorphous and crystalline phases was discovered in the 1960s [38], the involvement of chalcogenide alloy into the memory realm did not become popular until the advent of a new class of materials along the GeTe-Sb_2_Te_3_ pseudo-binary line [39–42], as shown in Figure 4. A fast switching and strong electrical/optical contrast can be found in chalcogenide alloys along this line with compositions (GeTe)_m_(Sb_2_Te_3_) where Ge_2_Sb_2_Te_5_ has been extensively implemented for various memory devices including DVD, PRAM, and SPPCM, and its physical properties were the most studied. Compared with materials located in the GeTe-Sb_2_Te_3_ pseudo-binary line, the phase-change devices that take advantage of undoped [43] and slightly Ge-doped materials with a composition of Ge_15_Sb_85_[44], and doped SbTe compounds such as In_x_(Sb_70_Te_30_)_1-x_ and Ag_x_In_y_(Sb_70_Te_30_)_1-x-y_ [45,46], usually allow for a faster switching speed due to different crystallization mechanisms. Another set in the pseudo-binary line along GeTe-Sb, represented by Ge_2_Sb_1_Te_2_ [47] and other alloys with the further addition of Ge was believed to provide very high thermal stability of the amorphous phase, thereby making it suitable for high-temperature memory applications [48]. The last group in Figure 4 belongs to Te-based eutectic alloys [49], which give a highly stable amorphous phase at room temperature by sacrificing the crystallization rate.

**Figure 5.**
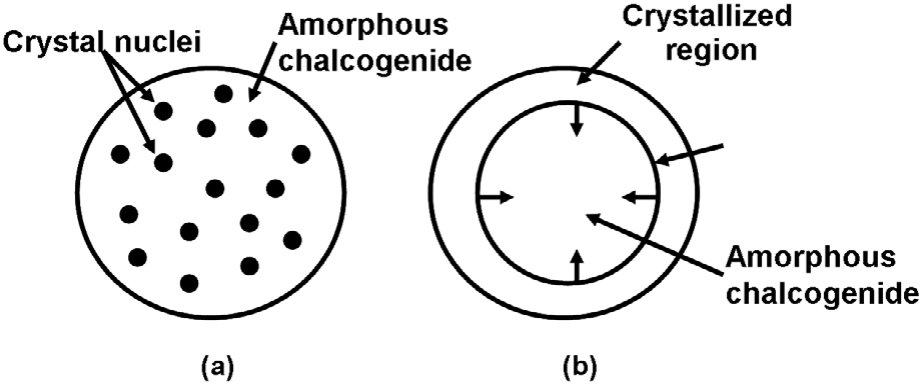
Crystallization kinetics of (a) nucleation-dominate and (b) growth-dominant materials.

It is evident that PCMs functionalize their storage operation through the switching of the physical properties between amorphous and crystalline states. The crystalline state where atoms display a long-range order is achieved by heating the amorphous state above the crystallization temperature while below the melting point, subsequently subjecting it to a slow cooling. Such a process is called crystallization. The incentive of the crystallization is commonly attributed to the classical nucleation-growth mechanism that small crystalline nuclei first are formed inside the amorphous matrix (i.e. nucleation), followed by the subsequent growth of phase front separating the amorphous and crystalline regions (growth). Accordingly, depending on their crystallization kinetics, chalcogenide alloys are usually classified into nucleation-dominated materials and growth-dominated materials. The crystallization kinetics of nucleation-dominated materials that are mainly located in the GeTe-Sb_2_Te_3_ pseudo-binary line are determined by the formation of nuclei and their consequent expansion [50], while growth-dominated materials that fall into the undoped and doped SbTe alloy relate crystallization to the expansion of the crystalline-amorphous interface [51], resulting in a faster crystallization rate particularly for high-scaled memory devices with small amorphous regions. Such a difference is schematically shown in Figure 5.

**Figure 6.**
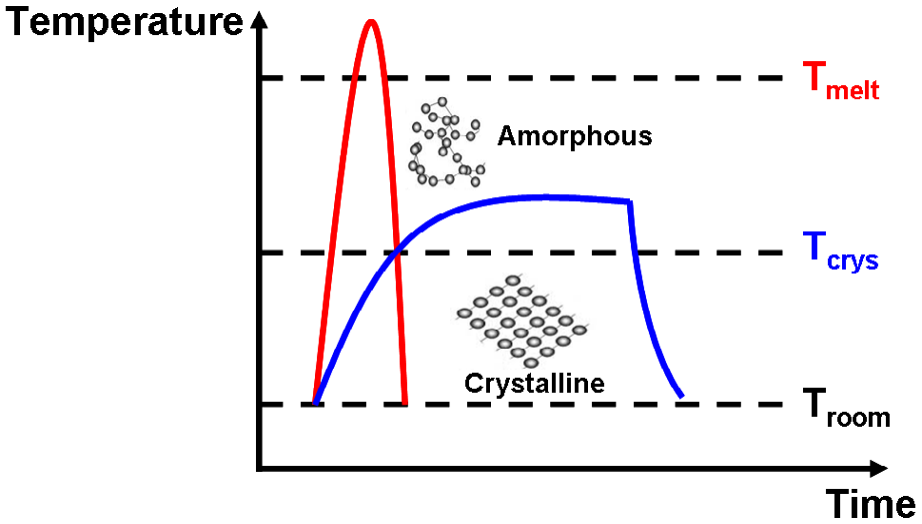
Crystallization and amorphization processes along with the temporal evolution of temperature.

The conventional scheme to induce amorphization where atoms are arranged in a short-range order is to heat crystalline materials above melting temperature and to quickly quench it to room temperature. Such a scenario associated with the aforementioned crystallization principle is depicted in Figure 6. No doubt this rapid annealing process generates numerous dislocations or vacancies causing a high resistance state inside the programmed region. Recently a novel ultra-fast photo-switching approach was proposed to achieve non-thermal amorphization via the rupture of the resonant bonds that inherently appear in the crystalline phase by applying ultrashort optical pulses [52], thereby inducing the amorphous phase without melting the materials. Based on this technique, the writing power for the required amorphization can be significantly reduced. In addition, another non-thermal amorphization mechanism was found on the phase-change based nanowires where the dislocations inside the crystalline phase move in the direction of the electric field [53,54], gradually accumulate in the nanowire, and finally result in jamming. This would cause a drastic increase in resistance and eventually induce the crystalline-to-amorphous phase transition. In spite of these innovative technologies, a detailed understanding of the amorphization and damage mechanisms governed by nonthermal processes is still lacking.

**Figure 7.**
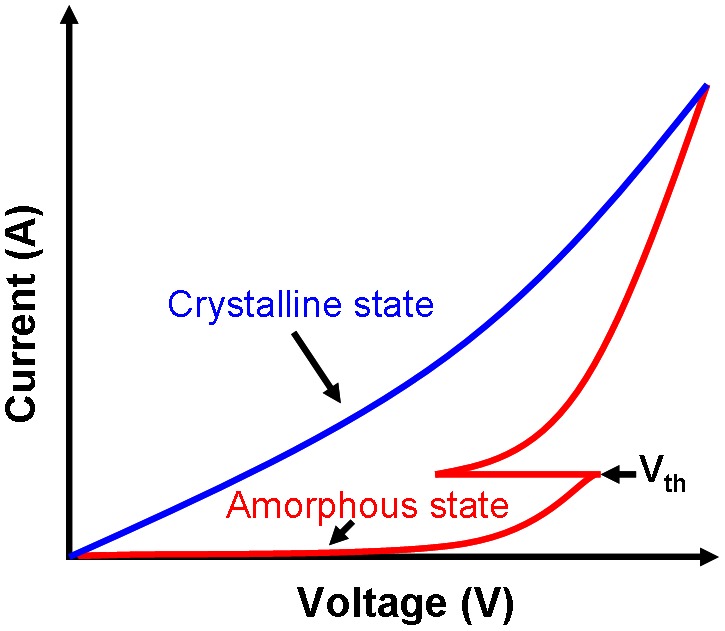
‘Threshold switching’ phenomenon.

The large variation on electrical properties of the PCMs, particularly on electrical resistivity, readily renders PCMs highly prospective candidates for storage applications. However, in contrast to the clear electrical conduction mechanism of the crystalline chalcogenide, the conduction mechanism of the chalcogenide in the amorphous phase still remains mysterious, mainly due to the well-known ‘threshold switching’ behaviour. It has been experimentally found that a sudden decrease in the electrical resistivity of the amorphous PCMs takes places when the applied electrical field is above a ‘threshold’ value, which is called ‘threshold switching’, as depicted in Figure 7. Although several theoretical models have been proposed to interpret the physics behind ‘threshold switching’, its mechanism still remains unclear. ‘Threshold switching’ was first attributed as being purely thermal-induced [55], as early observations revealed that the electrical conductivity of the amorphous materials increases along with the temperature. However, considering that the speed of switching is faster than the thermal time constant, this speculation was soon proved to be unreasonable, and in this case, ‘threshold switching’ is more ascribed to electronic mechanism than thermal effect [56,57]. Based on this, an impact ionization model owes the ‘threshold switching’ to the secondary carrier generation in the amorphous region [58, 59], while another electronic model explained ‘threshold switching’ as a result of energy gain of electrons in a high electric field giving rise to a voltage–current instability [60,61]. Differing from the above two methods, a field-induced nucleation model attributed ‘threshold switching’ to the formation of crystalline nuclei inside the amorphous region induced by the electric field [62]. As material parameters and device dimensions vary significantly within these three models, further experimental demonstrations as to which of the above models correctly explains the physics behind the ‘threshold switching’ are required. The electrical conductivity of PCMs in the crystalline phase is much simpler than the amorphous case, as it exhibits Ohmic/non-Ohmic behaviours at low voltage/high voltage bias respectively [58]. The Ohmic conduction can be attributed to the drift diffusion, whereas the thermal outcome that causes temperature-dependent resistance accounts for the non-Ohmic behaviour.

**Figure 8.**
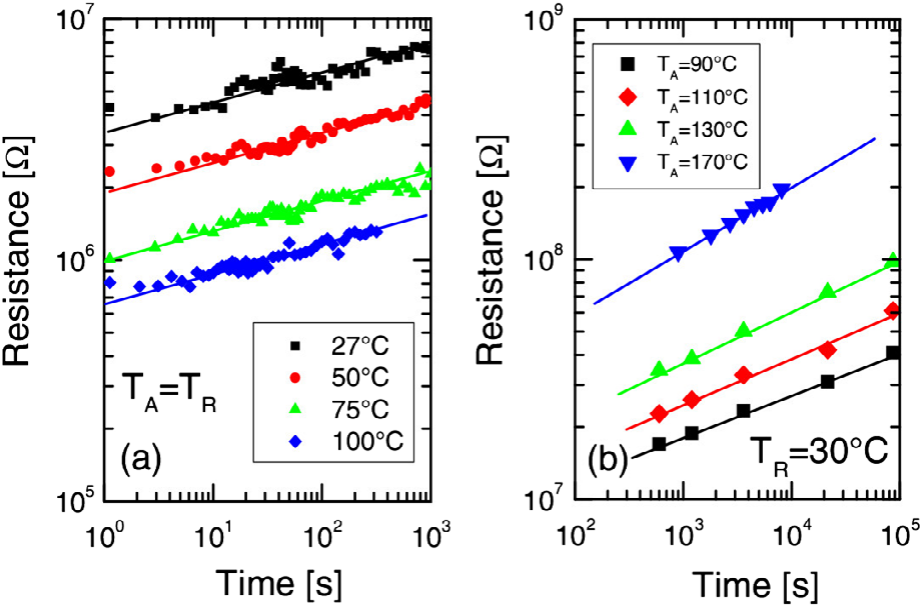
Measured resistance as a function of time for different annealing temperatures T_A_, under conditions T_R_ = T_A_ (a) and T_R_ = 30°C (b). Note the different temperature behaviour of the curve slope, indicating the power-law exponent ν of resistance drift. Reprinted with permission from [64].

In addition to ‘threshold switching’, another important electrical property of PCMs is the resistance drift that results in an increase in amorphous state resistance as a function of the time, following a power law R = R_0_ × (t/t_0_)^v^ where R and R_0_ are the present and initial resistances, t and t_0_ are the present and initial times, and v is an exponent that indicates the power-law slope [63]. As can be seen from Figure 8, the resistance of the amorphous state not only depends on the time elapse, but on the operating temperature as well as the volume of the amorphous region [64–66]. It is evident in Figure 8 that a larger drift exponent can be achieved using higher temperatures and larger amorphous volumes. Note that for conventional binary storage, higher resistance drift is desired to increase the On/Off contrast ratio. However, this would deteriorate the multi-level storage application [67], since different resistance drifts would finally merge with each other and consequently become indistinguishable. Similar to ‘threshold switching’, the cause of the resistance drift is also unclear at present, but it is suspected that it is induced either by a structural relaxation effect [63,68] or by mechanical stress relaxation [69]. Based on the structural relaxation model, the number of structural defects such as vacancies and distorted bonds are reduced and this results in an increase of the band gap and the activation energy for conduction while decreasing the trap densities [63,68]. Consequently, the trap spacing is extended to raise the barrier for conduction through the trap states, thereby causing the resistance drift. The mechanical stress relaxation is mainly due to the relaxation of the compressive stress developed in the amorphous phase in solidification stemming from the density variation between the amorphous and crystalline phases [69]. Although these two hypotheses can both fit the experimental observations well, there is still a lack of comprehensive study to prove their respective physical validity. In spite of the uncertainty about the origin of the resistance drift, an effective approach to suppress the resistance drift has recently been proposed by doping GeTe with an appropriate amount of either oxygen or nitrogen. It was reported that using either N-doped or O-doped GeTe can minimize the change of film thickness and mass density upon crystallization, thereby attenuating the stress caused by the volume change and leading to a reduced resistance drift [70,71].

The fact that crystallization and amorphization mechanisms are mainly governed by temperature undoubtedly indicates the importance of the thermal properties of the PCMs. Note that in order to achieve the required temperature inside the phase-change memory devices, particular attention needs to be paid to the thermal resistance arising from carrier energy scattering in the bulk material and the thermal boundary resistance (TBR) depending on scattering at the interface [72], which are strongly related to the thermal conductivity of the PCMs that can be measured by various techniques such as the 3ω method [73], picosecond time domain thermoreflectance [74,75], and nanosecond transient thermoreflectance [76]. The thermal conductivity of PCMs, such as different phases of Ge_2_Sb_2_Te_5_, ranges from 0.14 to 1.76 W/(m·K), while an even lower value of 0.05 W/(m·K) was obtained from high density nanostructured materials [77]. It is evident that such low thermal conductivity can significantly reduce the required power consumptions for a given temperature when compared to the higher thermal conductivity materials. In addition to the PCM itself, the selection of the electrode materials, mainly for devices that make use of Joule heating, also plays a critical role in determining thermal conduction, as an electrode with larger thermal conductivity than PCMs would cause dramatic heat loss. Under these circumstances, the electrode materials with relatively low thermal conductivity such as C and W-WN_x_ [78] are favoured over the conventional TiN electrode [79]. In addition to low thermal conductivity, electrode materials are also required to have high electrical conductivity to induce sufficient Joule heating for phase transformation at low programming currents. However, the requirements for materials to simultaneously exhibit high electrical conductivity and low thermal conductivity seem to be contradictory.

## 3. Tertiary memory using PCMs

### 3.1. Phase-change optical discs

The discovery of the GeTe-Sb_2_Te_3_ pseudo-binary line triggers the application of PCMs to the tertiary memory field which can be dated back to the invention of DVD RAM in the 1990s [80], and subsequently led to the temporary prosperity of phase-change optical disc families including rewritable CDs, rewritable DVDs, and Blu-ray discs [81].

Optical discs consist of a phase-change layer sandwiched by two dielectric layers that are usually made of ZnS-SiO_2_, which are deposited on a polycarbonate substrate. For recording purposes, a laser beam emitted from a semiconductor laser diode is propagated towards an objective lens by a beam splitter, and consequently focused onto the disc to form a laser spot. Therefore, the temperature inside the region bounded by the laser spot suffers from a rapid increase and induces the required phase transformation at the corresponding temperature. To read the previously written bit, the reflected light collected by the objective lens is sent towards the detectors through a beam splitter, which produces the readout signal and servo signals for automatic focusing and tracking. Due to the large optical contrast (mainly on reflectivity) between the amorphous and crystalline phases, the intensity of the reflected light obtained from the written bit differs significantly from the background material, thus readily distinguishing the phase transformed region from the untransformed background. According to the above descriptions, the storage capacity of the optical disc using PCMs mainly relies on the size of the laser spot whose diameter can be interpreted as a function of λ/NA where λ is the laser beam wavelength and NA is the numerical aperture of the objective lens that is given by nsinθ with n representing the refractive index and θ indicating the half-angle encompassed by the focused cone of light at its apex. Obviously either reducing the wavelength or increasing the numerical aperture can theoretically boost the storage capacity of the optical discs, leading to the debut of DVD RAM with a wavelength of 650 nm and numerical aperture of 0.85 [80,82] and Blu-ray with a wavelength of 405 nm and numerical aperture of 0.85 [81,83]. However, the feasibility of continuously scaling the wavelength and numerical aperture for higher capacity is severely handicapped by some insurmountable obstacles. On one hand, further decreasing the wavelength would inevitably require an appropriate ultra-violet laser source for optical recording that is however limited by current solid state laser technology and is not viable for practical use in the short term [84]. On the other hand, the substrate thickness needs to be decreased for a larger numerical aperture so as to sustain the tilt margin that varies inversely with the higher powers of the numerical aperture [85]. Because of this, achieving a numerical aperture greater than 0.85 usually accompanies a very thin substrate thickness down to 0.1 nm, rendering a system with high sensitivity to dust and fingerprints. Additionally, a higher numerical aperture would bring about a short depth of the focus that is determined by *λ* /NA^2^. Due to the aforementioned discussions, state-of-the-art optical storage technology using PCMs is restricted to a 405 nm wavelength and 0.85 numerical aperture, also known as the diffraction limit.

**Figure 9.**
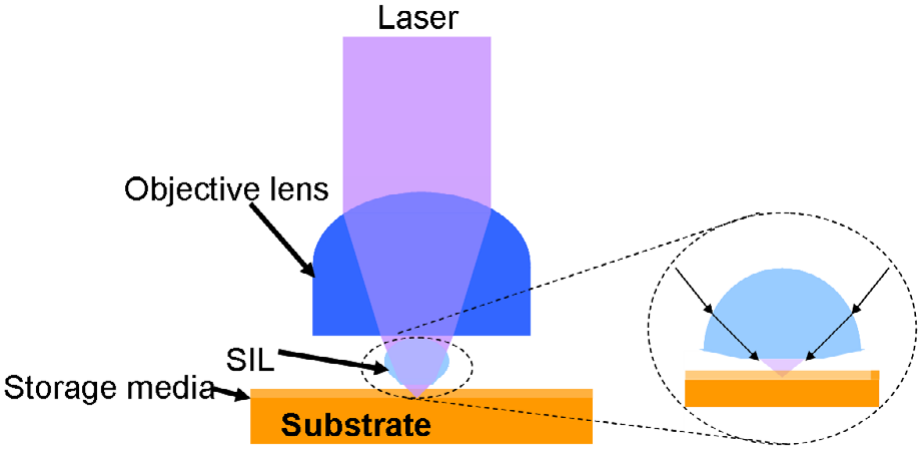
Optical near-field storage system.

In order to overcome the diffraction limit and thus augment the storage capacity of the optical disc, several alternative technologies that mainly include near-field optical recording [86–88], multi-layer recording [89–91], and multi-level recording [92–94], have been proposed and subject to deep study to facilitate the performance of the conventional far-field optical recording. The principle of the near-field technique is to insert a solid immersion lens (SIL) with high refractive index between the objective lens and the storage media to increase the numerical aperture, as shown in Figure 9. In principle, the surface of the SIL where light passing through a conventional objective lens is converged coincides with the focal plane of the laser beam. Nevertheless, in order to achieve the storage, the focal plane needs to be located on the recording media, and this results in a very short gap distance of less than 30 nm between the SIL and media to allow for evanescent wave coupling that can transfer laser energy from the SIL to the disc [86]. As the recording capacity of the optical disc based on the near-field technique strongly depends on the precise control of the gap distance between the SIL and disc, the ability to dynamically maintain the gap distance below 30 nm for a rotational, non-ideal plastic disc when encountering disturbances such as disc vibration and external physical shock becomes extremely critical [95–97]. As a result, a variety of advanced gap servo control technologies including feedforward control with zero phase error tracking method (ZPET-FF) [98], acceleration feedforward controller (AFC) method [97], and repetitive control method [99], have been assessed to improve the mechanical performance of the optical near-field system. Recently, an innovative near-field system with reducing harmonics of an axial run-out disturbance-feed-forward control (RHD-FFC) was proposed to show a precise gap servo for a 100 GB disc at 11,000 rpm [100]. It is essential to point out that the spot size can be further scaled down by coating an extra cover layer with a similar refractive index to the numerical aperture of the SIL over the data layer. Accordingly, an optical near-field system using a high refractive index cover layer and a SIL with a numerical aperture of 1.85 has been successfully fabricated [101]. Integrating this high numerical aperture technology with the advanced SIL precise gap servo systems would make it possible for near-field optics to break through the conventional diffraction limits.

Besides the near-field optics, some other technologies such as multi-level and multi-layer storage approaches have recently been subject to widespread research due to their potential for capacity growth. The fundamental of the multi-level technology arises from the fact that the reflectivity of the phase-change materials can vary along with its volume ratio of crystalline to amorphous regions. Hence, in contrast to single-level optical recordings that can store only 1 bit per recorded mark, multi-level technology enables the storage of multiple bits per recorded mark that are indicated by multiple reflectivity values obtained from continuous change of the crystalline to amorphous volume ratio. Via this technique, it is possible to increase the data capacity of the optical disc by a factor of log_2_(n) (n denotes the number of the level) [102]. Multi-layer technology follows a different path towards high storage capacity from the multi-level method by storing data in multiple layers of one disc, igniting research enthusiasm for the concept of 3D optical memory [89–91]. The future success of 3D optical memory to replace conventional optical discs fully depends on how closely these multiple layers can be packed but with minimum interlayer crosstalk [103], which still remains challenged with the use of PCMs.

Although today phase-change optical discs are still the most commonly used means for storing music, video, or data and programs of personal computers, it has always been in a relatively embarrassing position even since its birth, when compared with other phase-change technologies. This is mainly due to its much lower storage capacity and relatively high cost in contrast to HDD and NAND, and longer access time in comparison with DRAM and SRAM. Therefore, these disadvantages have ruled out the possibility of using phase-change optical discs for either main storage or secondary storage applications, while its long retention time and excellent portability renders the phase-change optical disc a promising contender for tertiary memory. However, the state-of-the-art storage capacity of the phase-change optical disc lags far behind the total amount of worldwide consumers’ archival data, and the recent development of cloud storage and media-on-demand devices even exacerbate its existing environment in the tertiary storage market. More importantly, its original realm for multi-media storage is being occupied by solid state drive (SSD) and on-line storage websites such as iTunes, Netflix, and YouTube that can provide better and friendlier access for consumers, whereby an optical drive is no longer essential hardware for both personal computers and laptops. As a result, the most common perspective with regard to the prospect of the optical disc is that it is gradually approaching its demise.

### 3.2. Scanning probe phase-change memory

**Figure 10.**
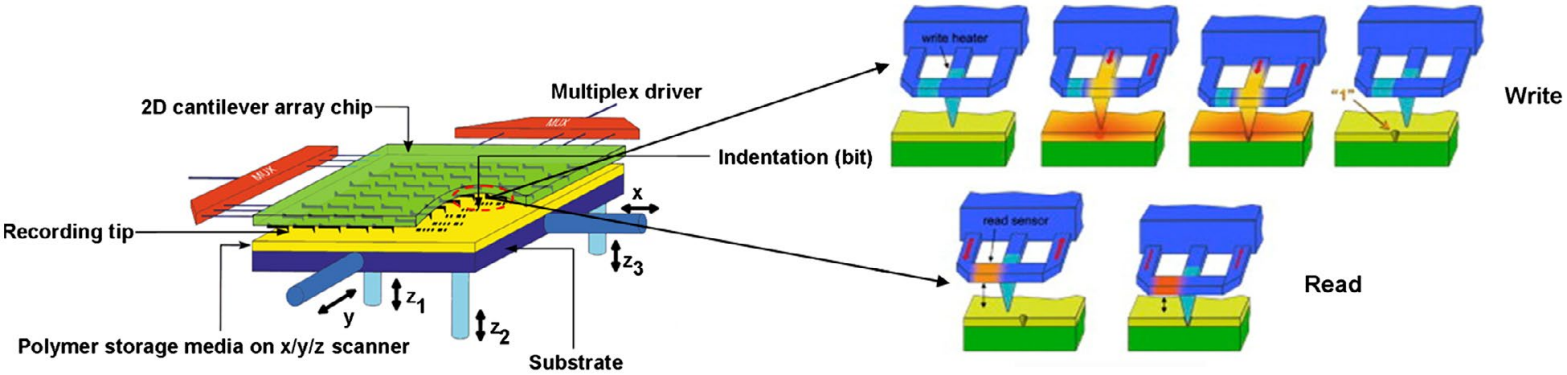
The ‘Millipede’ system when operated in its write and readout modes. Upgraded and reprinted with permission from [106].

As outlined in the previous section, the seemingly invincible capacity limit of the phase-change optical disc has therefore resulted in it excluding itself from the list of candidates for the future tertiary memory device, thus requiring the presence of more innovative technologies to rival the popular cloud storage. One representative of these advanced technologies is SPPCM that takes advantage of PCMs as storage media and a scanning electrical probe as the storage tool. The scanning probe, usually made of a cantilever with a sharp tip integrated on its end, was first used by atomic force microscopy (AFM) to map the tomography image of the sample surface that is interpreted as a function of the tip positions varying along with the atomic force between tip apex and sample surface [104]. AFM is usually operated in one of three image modes: contact mode, tapping mode, and non-contact mode [105]. During scanning, the tip is brought into contact with the sample surface for contact mode and tapping mode, while the tip is not in contact with the sample when operated in non-contact mode. It should also be noted that contact mode allows the tip to drag across the sample surface, while in tapping mode, the tip touches the surface only for a short time to prevent the tip from penetrating the sample which often occurs in contact mode. Due to the interesting features of AFM, it is possible to make use of AFM to change the physical properties of the sample surface especially when in contact with the tip, giving rise to the original idea for probe-based storage. However, the concept of probe-based storage did not become a popular research topic until IBM proposed their thermal-mechanical probe memory in 2002 [106–108], also known as the ‘Millipede’ system. As revealed in Figure 10, the recording of thermo-mechanical probe memory is realized by pulling a heated tip into the polymer medium to form an ‘indentation’ that indicates the binary bit, and the readout is performed by sensing the resistance variation of a read resistor with temperature-dependent resistance during the raster scanning. The combination of the nanoscale probe with polymer media obviously provides thermal-mechanical probe memory with several exceptional characteristics such as ultra-high recording density and fast switching speed; more importantly, the successful application of the thermo-mechanical probe on the storage field has raised the feasibility of using scanning probes to transform the phase of chalcogenide alloy, thereby resulting in the concept of SPPCM.

**Figure 11.**
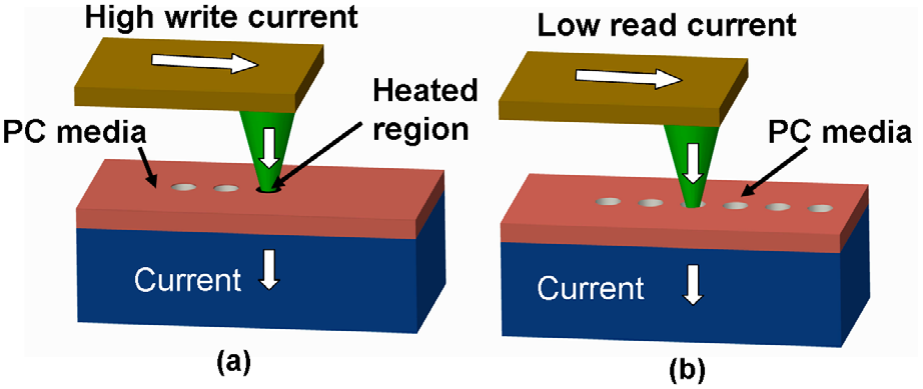
Phase-change probe memory when operated in (a) write mode, and (b) read mode. Reprinted with permission from [17].

The physical principles of SPPCM for both recording mode and readout mode are schematically shown in Figure 11. During the recording process, a writing current flowing through the conductive cantilever and the tip is injected into the phase-change layer when it is brought into contact with the tip, which enables the Joule heating inside the phase-change layer to switch the phase between crystalline and amorphous states once the required temperature for phase transition is reached. Readout is executed by applying a relatively low electric potential into the phase-change layer through a conductive tip during the raster scanning. Due to the remarkable resistance difference between the crystalline and amorphous regions, the resulting current obtained when the tip is on top of the crystalline region would be eminently distinct from that for the amorphous case. Because of this, the phase transformed region can be easily distinguished from the untransformed background. There is no doubt that due to the implementation of PCMs as the storage media, SPPCM consequently inherits the advantageous characteristics of the PCMs such as fast transition and superb stability associated with large electric/optical contrast [109]. Moreover, adopting the nanoscale-sized tip as the storage tool would enable SPPCM to confine the phase transformation occurring in a nanoscale region with a similar size to the probe tip, thereby sharply increasing the resulting areal density [19]. Such a small phase transformed region in conjunction with an exemption from the use of the thermal probe can effectively lower the required power consumption in comparison with the ‘Millipede’ system. Thanks to these merits, SPPCM is considered to be the most promising candidate for next-generation tertiary memory and archival storage devices, and the correlated recording and readout experiments based on various SPPCM architectures have therefore been performed by global researchers to exploit the ultimate potential of SPPCM for tertiary storage applications [110–118]. However, according to these reports, the diameters of the generated phase-change marks either in the crystalline or amorphous phase vary from 20 to 80 nm, giving rise to an areal density much lower than the desired values. This is most likely due to the relatively superficial understanding of the key factors affecting the operation of the SPPCM as well as the lack of systematic analysis and optimization of the SPPCM structure to continuously promote its recording/readout performances. In this case, a deep insight into the roles of the storage stack and probe tip of SPPCM on its physical operation principles would greatly benefit further density enhancement.

**Figure 12.**
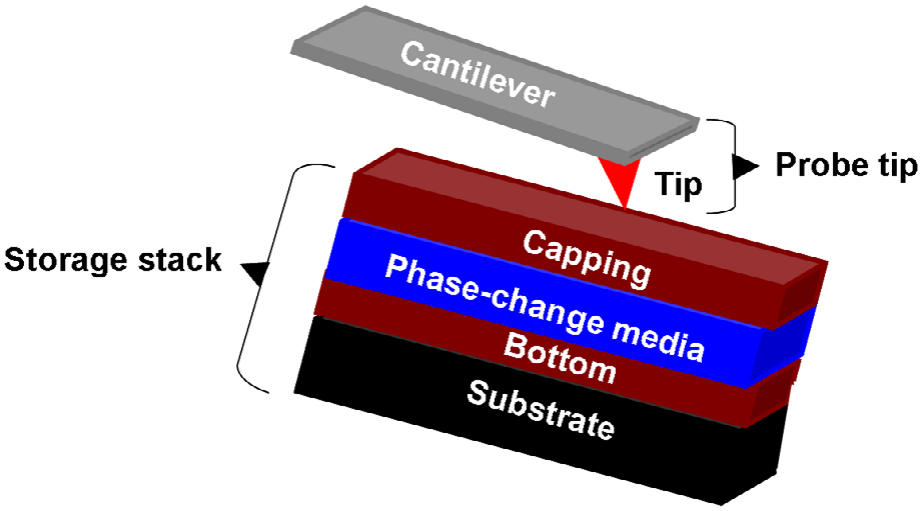
The typical architecture of SPPCM.

The SPPCM device is usually divided into the storage media stack and the probe tip, illustrated in Figure 12. The storage media stack is composed of a phase-change storage layer sandwiched by a capping layer and a bottom layer, which are deposited on a substrate. The role of the capping layer is to protect the phase-change layer from oxidation and wear as well as providing a proper electrically and thermally conductive path for writing current and readout potential [17]. To meet the latter, a thin capping layer with intermediate electrical conductivity and low thermal conductivity is preferable to provide sufficient Joule heating with a small programming current [119]. It should be also noted that the capping layer cannot be too conductive, as this would cause a short circuit particularly when reading amorphous marks from a crystalline background [120]. Considering this, diamond-like-carbon (DLC) has been widely considered to be the main constituent for the capping layer due to its high mechanical hardness and its flexibility to adjust the related physical properties [121]. As the storage layer, the phase-change layer is made of the chalcogenide alloy (primarily Ge_2_Sb_2_Te_5_) whose phase can be transformed between amorphous and crystalline states by the Joule heating. To induce the required temperature, the thickness of the phase-change layer needs to be thin to reduce the threshold voltage [119]. The bottom layer usually acts as an electrode to collect either the writing or readout current and also behaves like a thermal insulator to overcoming possible heat diffusion through the substrate that is commonly made of Si with high thermal conductivity [119]. As a result, the bottom layer is required to have high electrical conductivity with low thermal conductivity in comparison with the capping layer [122]. This seemingly contradictory property requirement makes the search for the appropriate electrode material somewhat difficult and accordingly DLC media has been previously adopted as a trade-off. However, a recent study has successfully applied the TiN electrode previously employed in a PCRAM device into the SPPCM to replace the DLC electrode [123], and because of the excellent compatibility of TiN with high electrical conductivity and low thermal conductivity, the resulting energy consumption from writing a crystalline mark using TiN at the bottom is at least one order of magnitude lower than the device with a DLC electrode at a similar mark size [123]. This finding clearly indicates the favourite position of a TiN electrode over DLC for the design of future SPPCM devices.

**Figure 13.**
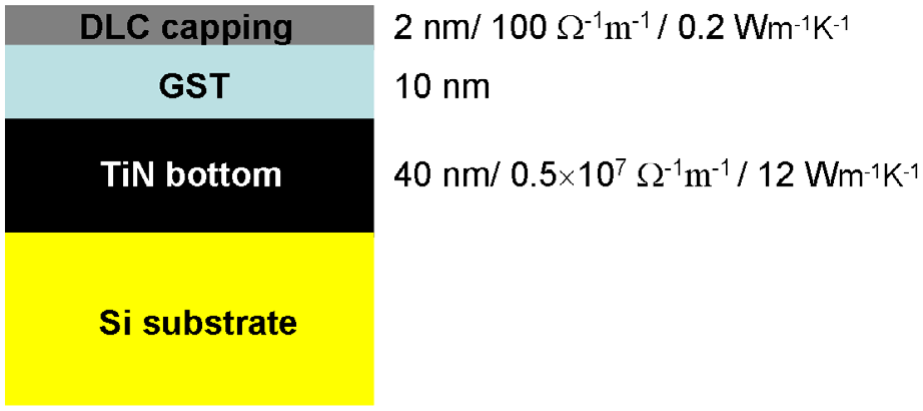
The designed optimized storage stack for SPPCM.

After establishing the materials for each layer of the storage stack, it is vital to optimize the thickness and the physical properties of each layer in the stack to offer the best physical performances, and thus to create a design criterion. To achieve this goal, a parametric electro-thermal model has been developed that comprises a time-resolved Laplace and heat conduction equations for describing electrical and thermal processes, respectively. The model was used to calculate the maximum temperature inside the phase-change layer as a function of the thickness and the electrical/thermal conductivities of each layer inside the storage stack, i.e. the DLC capping layer, Ge2Sb2Te5 (GST) layer, and TiN bottom layer, for different programming currents [110]. The aim of introducing such a method is to find out the optimal layer thickness and electrical/thermal properties for a storage stack thus to allow for the formation of ultra-small written marks (i.e. ultra-high density) at the cost of ultra-low energy consumption, resulting in an optimized SPPCM architecture shown in Figure 13.

**Figure 14.**
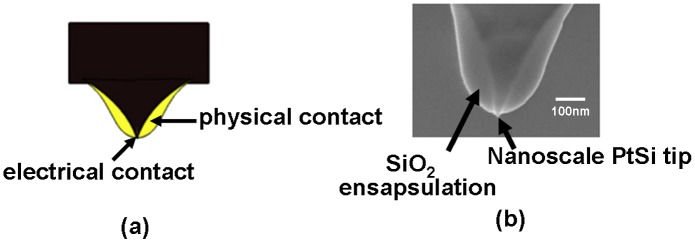
(a) Schematic of the ‘encapsulated’ tip concept, and (b) the experimentally fabricated encapsulated tip. Upgraded and reprinted with permission from [124].

In addition to the storage stack, the physical properties of the used probe tip can also strongly affect the write and readout performance of the SPPCM. Acting as a conductive bridge towards the storage stack, the electrical conductivity of the probe tip needs to be high to reduce the voltage drop across the probe, whereas it must also have low thermal conductivity to suppress the heat escaping from the probe to the surrounding air [119]. Most importantly, the fact that the probe tip would frequently move back and forth over the sample surface can deteriorate the issue of tip wear due to the combinations of the high atomic force between tip and capping layer and the used hard sample associated with the high current density at the tip apex. In this case, severe tip wear would increase the tip diameter and in turn reduce the areal density. For SPPCM, the tip diameter needs to be maintained below 10 nm for storage densities of 4 TB in^−2^ and higher after a sliding distance over several kilometres [124], which is a rather challenging requirement. In this case, it is not possible to directly reproduce the presently available commercial probes that do not address the long-term reliability of the tip apex for sustained conduction for SPPCM applications, and attention has been switched to the use of hard materials that can preserve the nanoscale integrity of the tip apex over long sliding distance. Platinum silicide (PtSi) is one type of promising hard material that has a superior anti-wear characteristic [125], whereby forming PtSi at the tip apex can significantly mitigate the wear property of the tip. Additionally, the highly electrically conductive attribute of PtSi renders the probe tip a far superior nanoscale electrical contact capable of sustaining fairly high current densities. Another effective approach to alleviate tip wear is to encapsulate the conductive probe core with some dielectric materials [124]. This idea arises from the common-sense view that increasing the tip diameter can enlarge the area between tip and sample, which in turn reduces the pressure that the tip suffers from. Note that increasing the tip diameter does not sacrifice the resulting areal density that is mainly determined by the electrically conductive tip diameter (i.e. the diameter of the conductive core) rather than the physical tip diameter. Therefore, the combination of forming PtSi at the tip apex with a dielectric encapsulation of the conductive probe core leads to the concept of a SiO_2_ encapsulated Si tip with PtSi at the tip apex [124,126], as illustrated in Figure 14, which was successfully fabricated by IBM in 2009. The capability of using this encapsulated conductive tip to maintain a long-term electrical conduction for at least 2.5 m of sliding has been demonstrated [124], guaranteeing its suitability for SPPCM.

Based on the above analysis, the optimized SPPCM is to be regarded as an integration of the storage stack shown in Figure 13 with a SiO_2_ encapsulated Si conductive core with PtSi at the tip apex. As a result, the write and readout performances of the proposed optimized SPPCM were re-assessed by the previous electro-thermal model but complemented with several advanced modelling techniques including a classical nucleation-growth equation for crystallization process [127], threshold switching effect based on the trap transport theory [60], electrical contact resistance at PtSi/DLC interface [128], and TBR at the interfaces of DLC/Ge_2_Sb_2_Te_5_ and Ge_2_Sb_2_Te_5_ /TiN [74], to take into account all the possible factors that could affect the physical properties of the SPPCM. It is also necessary to mention that the writing of an amorphous mark from a crystalline background has been experimentally proven to be difficult due to the required high temperature above the melting point that may oxidize the capping layer [129]. To the best of our knowledge, there is only one research group (Tanaka’s group from Hokkaido University) who have successfully achieved the writing of an amorphous mark that was obtained from a storage stack without the use of a capping layer [115]. Such a structure is not suitable for practical use as the lack of a capping layer would clearly bring several adverse effects such as wear and oxidation into the capping layer. Therefore, the majority of efforts made on SPPCM are currently concentrated on the writing of crystalline marks rather than amorphous marks.

**Figure 15.**
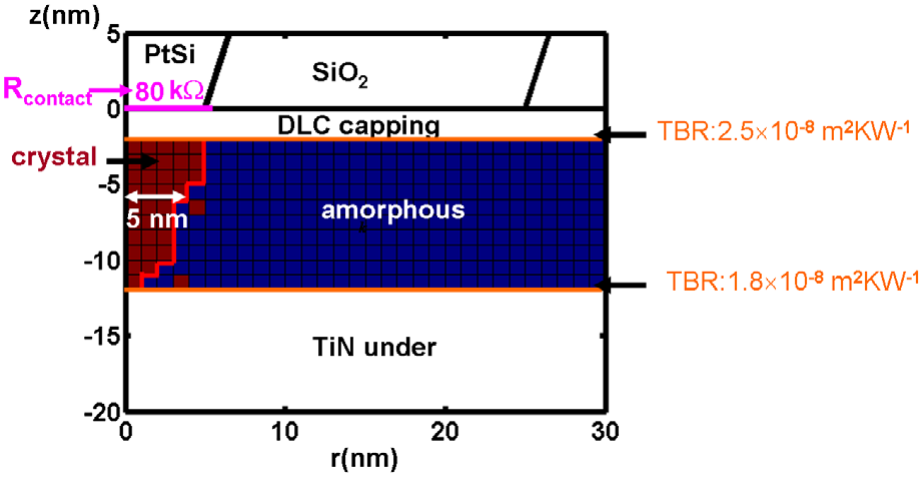
The resulting crystalline bit from the designed optimal electrical probe memory. The written bit exhibits a radius of approximately 5 nm, corresponding to 10 Tbit/in^2^. Reprinted with permission from [130].

The crystalline mark attained from the aforementioned comprehensively physical model using the designed optimized architecture is shown in Figure 15 [130]. As revealed from Figure 15, a crystalline bit with a diameter of ~10 nm extending through the whole amorphous layer was formed by a 6 V electric pulse of 100 ns, thereby allowing for various potentially attractive features for tertiary memory that include an areal density of 10 Tbit/in^2^, a data rate of 1 Mb/s, an energy consumption of 1.6 pJ, and a long retention time at archival temperature.

**Figure 16.**
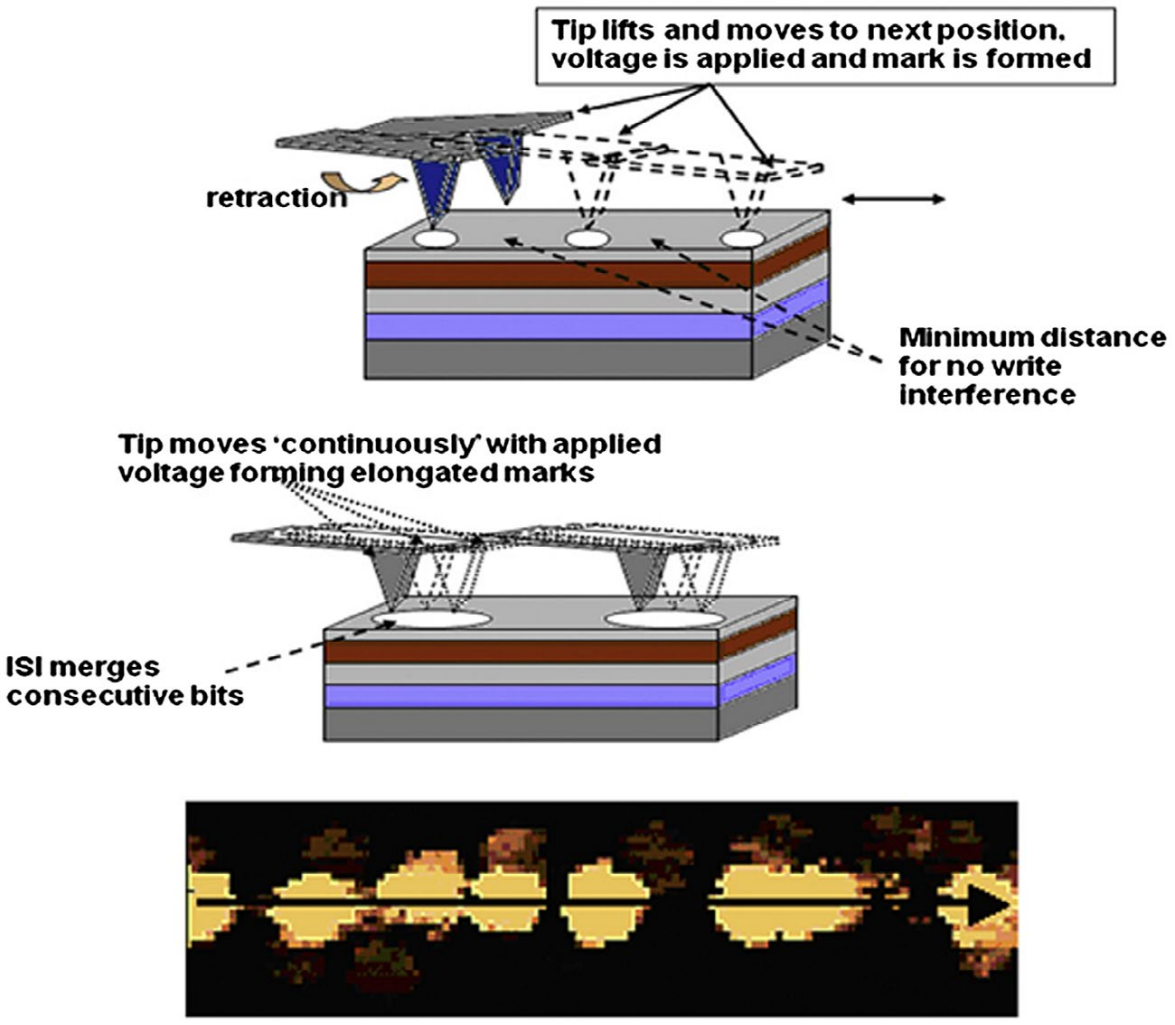
Schematic (top) of conventional mark-position recording, as used in probe memories to date; schematic (middle) of a new mark-length recording strategy; and (bottom) a current image of mark-length recorded bits in a phase-change medium (image is 580 nm × 140 nm and recorded bit sequence is 11001110111101110001111000111 with a bit cell length of 20 nm). Reprinted with permission from [131].

**Figure 17.**
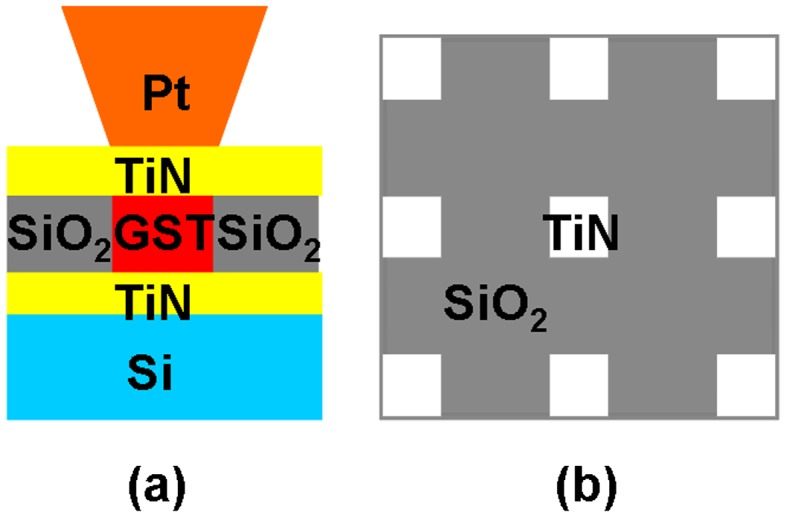
(a) Front view and (b) top view of SPPCM based on patterned PCMs.

Besides the conventional probe recording schemes introduced above, some advanced writing strategies for SPPCM have recently been under investigations so as to further enhance the competitiveness of the SPPCM against other storage devices, the most exciting of which is mark-length recording, also known as groove recording [131]. Differing from the conventional recording usually operated in the tapping mode, this novel technique drives a tip sliding a small distance over the sample surface to generate a recorded bar with an arbitrary length governed by the inter-symbol-interference (ISI) effect. Given this intriguing scheme, the adopted tip size is no longer directly related to the recorded mark length, consequently allowing for the possibility of achieving high areal density without the requirement for ultra-sharp tips. A comparison of the writing mechanism between groove recording and conventional recording is shown in Figure 16 associated with the images of written bits using the groove recording approach [131]. Another encouraging recording scheme stimulated by the recent progress of nanolithography technology is to make use of small patterned phase-change cells isolated from each other by a thermal insulator [132], clearly illustrated in Figure 17. The idea that ultra-high areal density can be secured through the phase transition occurring inside each small patterned phase-change cell has been demonstrated by a patterned cell with a 5×5 nm^2^ size separated by a 3 nm SiO_2_ gap [132], giving rise to a density up to 3.8 Tbit/in^2^.

## 4. Secondary memory using PCMs

**Figure 18.**
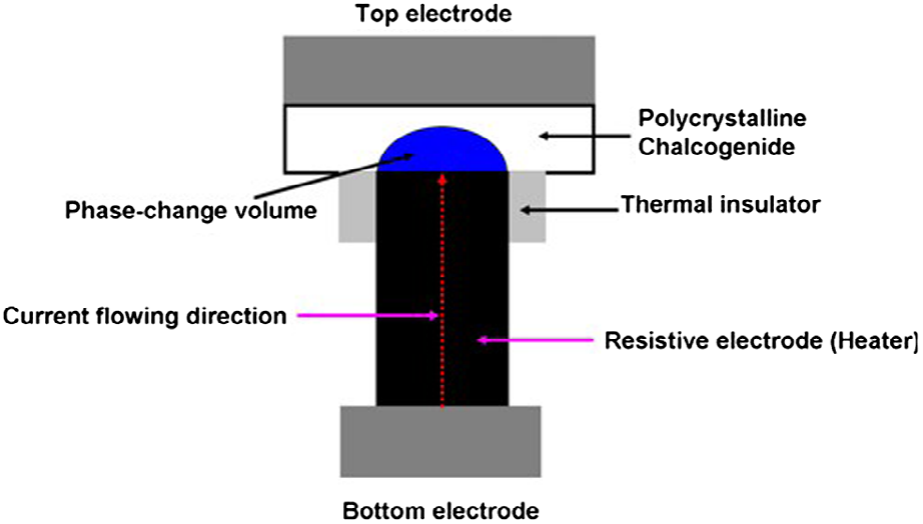
The cell structure of the OUM PRAM.

The application of PCMs to secondary memory storage led to the birth of PRAM that is undoubtedly the most prestigious phase-change memory to date, due to its prevailing characteristics for great scalability, fast switching speed, long retention time, and excellent endurance [133]. The history of PRAM can be traced to the concept of the Lance-like PRAM cell that became the root on which a variety of different PRAM structures with improved performance were designed [134]. The structure of Lance-like PRAM, known as ovonic unified memory (OUM), is schematically described in Figure 18. According to Figure 18, the OUM cell is made up of a phase-change layer sandwiched by a top metal electrode connecting to the external circuit and a resistive electrode usually called the heater, which are encompassed by a thermal insulator. Programming is performed by applying a high current from the bottom electrode to the top electrode via the heater, and a high current density stemming from the small interfacial contact area is induced at the phase-change layer/heater interface where the phase transition is formed according to the resulting Joule heating. Similar to SPPCM, readout is accomplished by detecting the current through the cell excited by a low readout voltage.

Using appropriate electric pulses would help PRAM to achieve either an amorphous phase from a crystalline background or vice versa. The amorphization process for PRAM operations is conventionally called ‘RESET’, while the crystallization process is named as ‘SET’. As amorphization process requires a temperature for melting; the ‘RESET’ pulse is accompanied by a high writing current that determines the power consumption of PRAM. However, ‘SET’ only requires a low voltage to reach the crystalline temperature due to the threshold switching effect that also reduces the self-heating in the amorphous region during low voltage readout, and so suppresses the READ disturbance [135]. The duration of the ‘SET’ pulse depends on the crystallization speed of PCMs and therefore dominates the write speed of the PRAM. The ability for PRAM to have a switching speed in the order of a few hundred nanoseconds [136,137] readily makes it satisfy the market demand for secondary memory applications. However, in order to outperform other mainstream storage devices such as HDD and NAND, devices with even faster switching speed and a high cycle numbers (10^8^–10^9^) are required, which imposes more rigorous demands for PRAM technology. Fortunately, these formidable challenges have been addressed by the recent development of new materials such as reactively sputtered and doped Ge_2_Sb_2_Te_5_ having the cycle numbers to 10^9^ while sustaining fast switching down to 20 ns [138,139]. Another important property of the secondary storage memory is data retention time that requires 10 years at 150°C for automotive applications [140,141] and tens of seconds at ~260°C for pre-coded chips that need to pass a solder bonding process [142]. To obtain much better data retention at higher temperature, one possible solution is to increase the crystalline temperature of Ge_2_Sb_2_Te_5_ through materials modification that in turn impairs the switching speed of the device [143–145]. Such a paradox remained a question until the advent of Ge_2_Sb_1_Te_2_ considered as the best compromising material located in a tie line between GeTe and Sb, whose thermal stability can be further improved by increasing Ge concentration [146]. The use of this highly Ge-rich Ge-Sb-Te alloy results in high crystalline temperatures at ~250°C, fast speed (80 ns), ~30% lower RESET current against Ge_2_Sb_2_Te_5_, 10^8^ cycling endurance, and tolerance to up to 190°C testing in a 128 Mbit phase-change memory test chip [146,147]. Additionally, recent findings reveal that incorporating the above highly Ge-rich composition with N or C dopants can lead to better thermal stability [148–150].

For PRAM to completely rule the secondary storage market in the near future, its scalability must last at least several generations so that investors can be confident in their continued commitment. However, scaling PRAM devices down to nanometre size would inevitably affect the device properties that in turn govern the ultimate device scalability. In this case, a deep understanding of the relationships between device properties and device scalability and the possible methodologies to scale device parameters at nanoscale size become of high importance.

**Figure 19.**
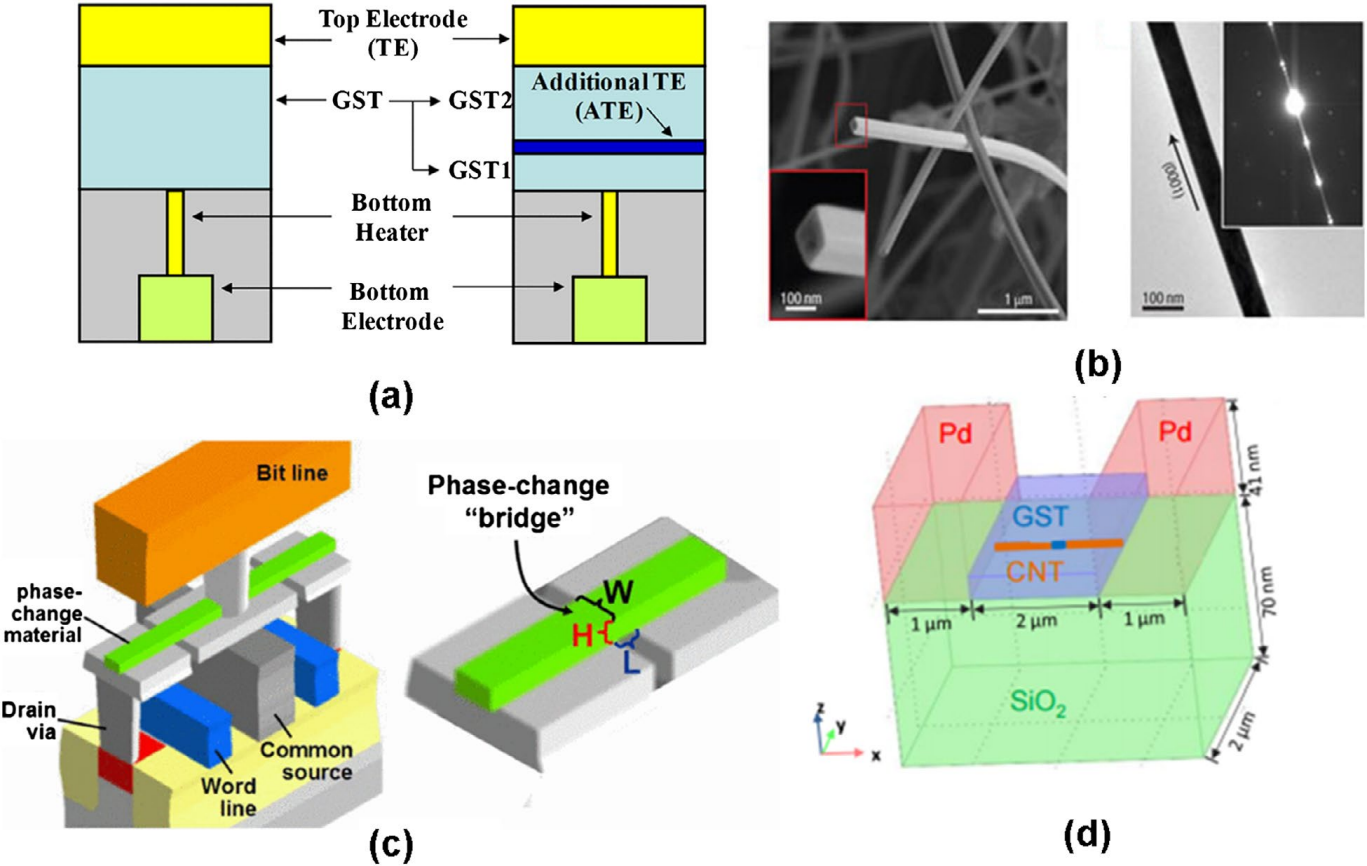
Various PRAM devices for threshold voltage scaling using: (a) additional electrode, reprinted with permission from [154]; (b) phase-change nanowire, reprinted with permission from [155]; (c) phase-change bridge, reprinted with permission from [44]; and (d) carbon nanotube (CNT) electrode, reprinted with permission from [160].

**Figure 20.**
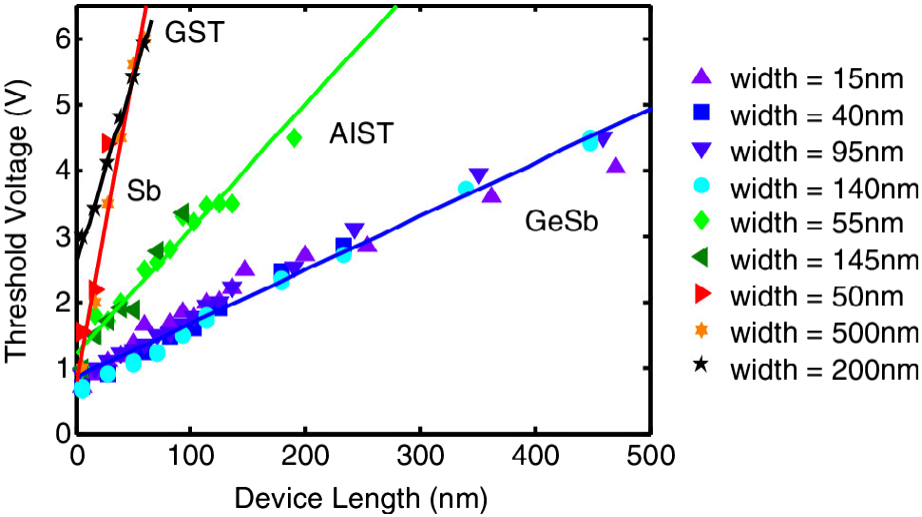
Threshold field as a function of the length of the active phase-change region in a phase-change bridge memory for four different PCMs. Reprinted with permission from [159]. ‘AIST’ refers to ‘Ag_4_In_3_Sb_66_Te_27_’.

The first major property significantly affected by device scalability is threshold voltage. As described above, threshold voltage is regarded as linearly proportional to the thickness of the phase-change layer. Although the ultimate scalability limit of the PRAM is yet to be established experimentally, the minimum layer thickness is theoretically estimated to be ~2 nm from the thermodynamic point of view [151,152]. If the aforementioned voltage-layer thickness relationship still applies to ultra-thin phase-change film, the threshold voltage will fall below ~0.1 V for < 5 nm thick phase-change layer. Such a small threshold voltage will become indistinguishable from readout voltage and thus fail the readout of PRAM as well as exacerbating the susceptibility of data bits to voltage fluctuations, causing unintended data loss [153]. Given the unclear mechanism of the threshold switching, the trend of the threshold voltage changing with the film thickness when downscaling to either the inter-trap distance or the minimum distance that the carriers have to travel is still vague. As a result, a number of PRAM cells with different structures have been investigated to study the influence of layer thickness on threshold voltage within an ultra-thin regime, giving rise to Figure 19. A commonly used approach, as described in Figure 19(a), is to add an extra top electrode into the phase-change layer at an arbitrary height from the bottom electrode to confine the programming volume to this arbitrarily defined thickness [154]. The resulting observation from this specific cell follows the previous finding that the threshold voltage is linearly proportional to the active layer thickness. However, the conventional scaling law that governs the threshold voltage changes from constant field to constant voltage scaling for the length of the amorphous region below 10 nm using nanowire phase-change devices [153,155,156]. Another alternative cell structure for threshold switching scaling is a phase-change bridge device consisting of a narrow line of thin phase-change material bridging two underlying electrodes [157, 158]. Such a bridge cell allows for measuring the threshold voltage as a function of the device length for different materials, whereby the threshold fields for Ge_15_Sb_85_, Ag-and In-doped Sb_2_Te, Ge_2_Sb_2_Te_5_ and 4 nm thick Sb devices are reported to be 8.1, 19, 56, and 94 V/μm, as illustrated in Figure 20 [159]. A novel PRAM cell with an active device area acting as a nanoscale gap between two carbon nanotube electrodes through electrical breakdown has recently been proposed [160,161]. By carefully controlling the breakdown voltage, the threshold voltage was found to change linearly with the size of the nanogap varying from 20 to 300 nm, giving rise to an average field of 100 V/μm for Ge_2_Sb_2_Te_5_.

**Figure 21.**
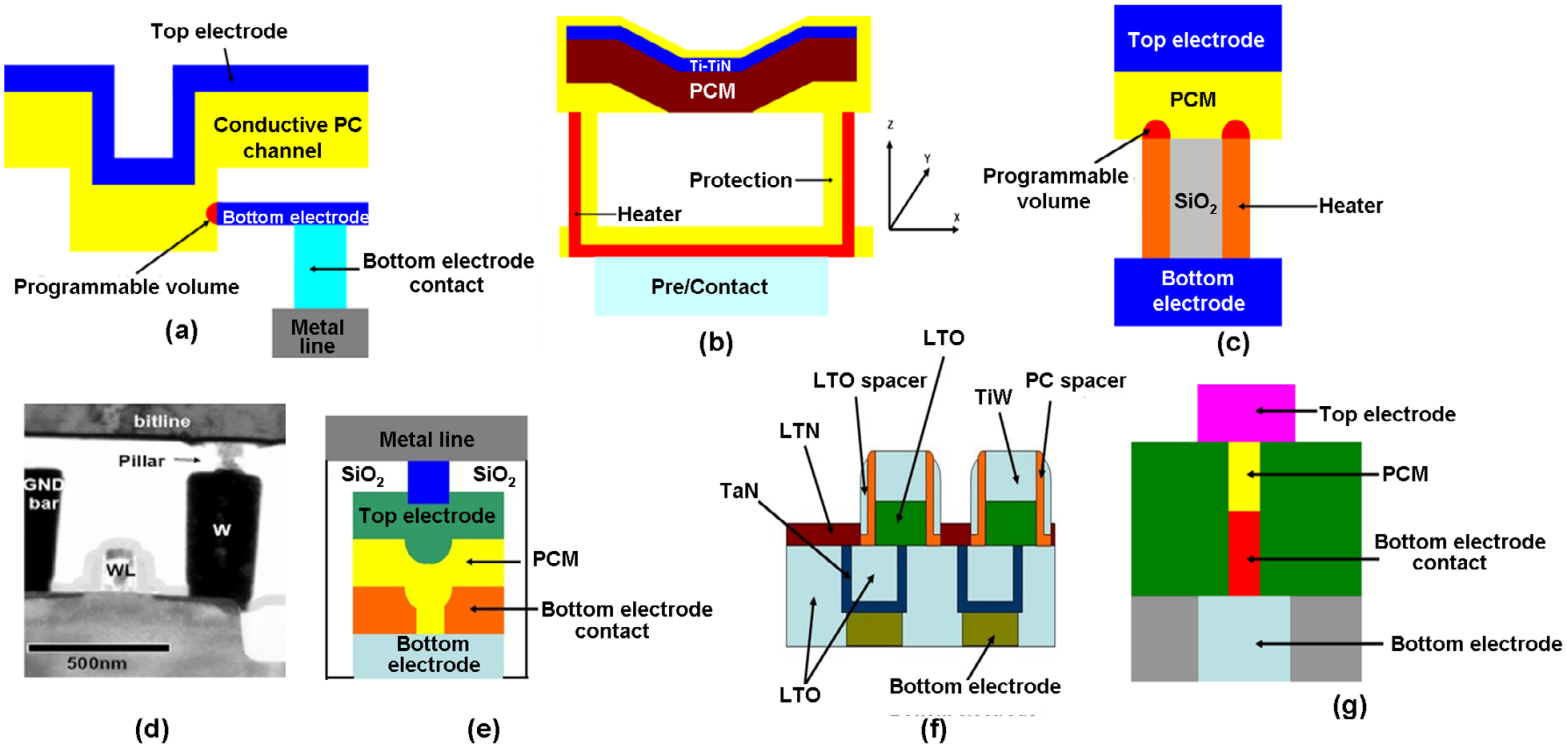
Various PRAM devices for ‘RESET’ current scaling using: (a) edge-contact type; (b) μTrench type; (c) ring-shaped type; (d) pillar type; (e) pore type; (f) cross-spacer type; and (g) dash type. Reprinted with permission from [162].

Besides the threshold voltage, the ‘RESET’ current is another important property that needs to be treated seriously during the scaling process of PRAM, as it dominates the energy consumption of the device. For PRAM application, the ‘RESET’ current is usually delivered by a cell selector connecting in series with PRAM and in this case the scaling of PRAM must accompany the scaling of the cell selector. However, so as to generate adequate ‘RESET’ current for the required phase transition, the size of the cell selector is forced to be maintained at a relatively large scale, thus severely limiting the scalability of the PRAM device. Therefore, the scaling of the ‘RESET’ current becomes critically important for PRAM to achieve ultra-high storage density and low power consumption. Conventional routes adopted to reduce the ‘RESET’ current were devoted to either reducing the contact area at the heater/PCMs interface or shrinking the programming volume, consequently resulting in numerous device structures such as edge-contact type [162,163], μTrench type [162,164], ring-shaped type [162,165], pillar type [162,166], pore type [162,167], cross-spacer type [162,168], and dash type [162,169], illustrated in Figure 21.

**Figure 22.**
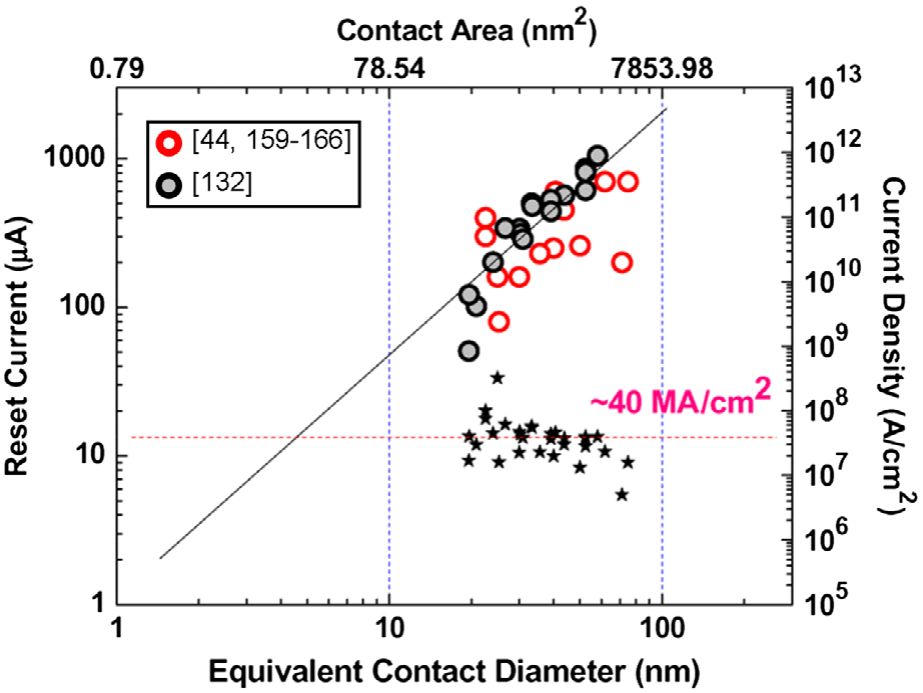
‘RESET’ current as a function of equivalent contact diameter, showing a linear scaling trend with the effective contact area as the device feature size goes down. A constant ~40 MA/cm^2^ current density is required to program the PRAM cell. Reprinted with permission from [14].

**Figure 23.**
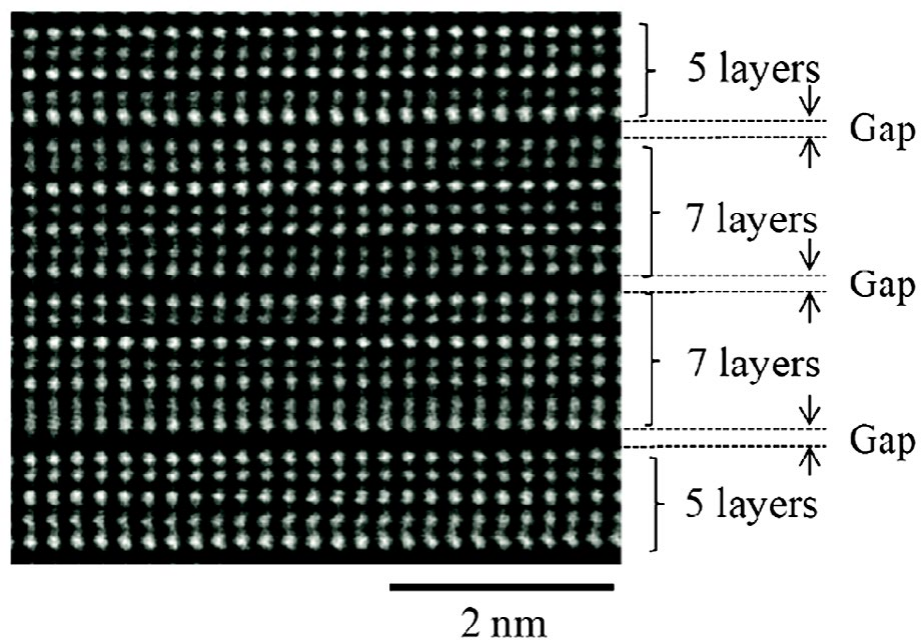
High-angle annular dark field (HAADF)-scanning transmission electron microscope (STEM) image of GeTe/Sb_2_Te_3_ superlatttice. Reprinted with permission from [170].

The presence of these innovative cell structures enables a remarkable reduction of the ‘RESET’ current along with the downscaling of the effective contact area: such a relationship is clearly reflected in Figure 22 for different cell structures. Accordingly, the ‘RESET’ current is proportionally reduced by decreasing the effective contact area, and a constant current density of ~40 MA/cm^2^ is required to program an average PRAM cell. Note that the required ‘RESET’ current density can be lowered even further by engineering the PRAM cell to sublithographic sizes with the introduction of spacers or the aforementioned carbon nanotube electrode materials, resulting in a current density down to ~10 MA/cm^2^ [16]. Differing from the above methods, a novel concept of an interfacial phase-change memory that makes use of a superlattice PCM stack formed by alternating two crystalline layers with different compositions, as illustrated in Figure 23, has recently attracted considerable attention [170]. According to the alignment of the c-axis of a hexagonal Sb_2_Te_3_ layer and the <111> direction of a cubic GeTe layer in a superlattice, Ge atoms can be switched between octahedral sites and lower-coordination sites at the interfaces between superlattice layers [170]. The advantages of this interfacial phase-change memory over conventional PRAMs arise from its ability to induce switching without melting materials [171]. The switching mechanism of interfacial phase-change memory is reported to be governed by the limited movement of Ge atoms with the conduction channel of Te-Te inducing the difference in resistance [171–173]. Due to its exceptional switching property, the ‘RESET’ current was confined down to 3 μA with a Sn_10_Te_90_/Sb_2_Te_3_ superlattice PRAM cell [170].

**Figure 24.**
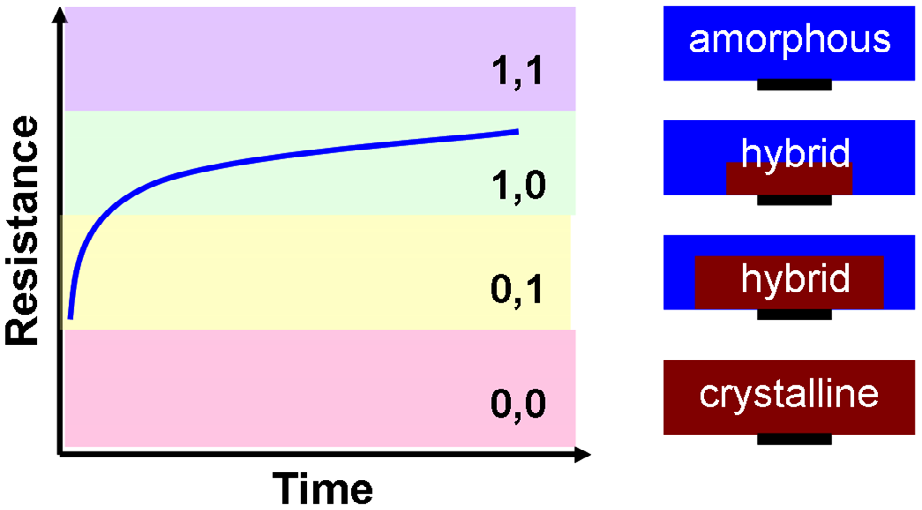
MLC for storing four logical states.

**Figure 25.**
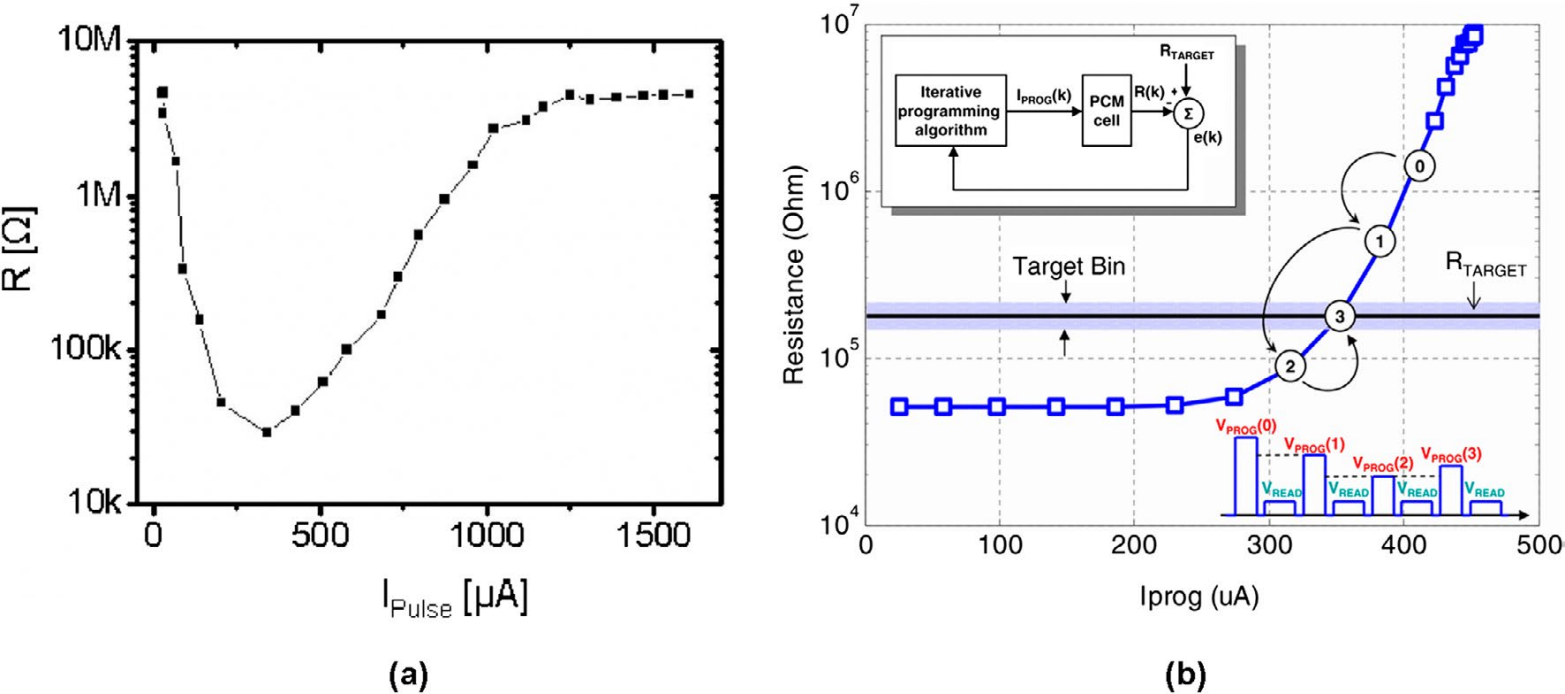
(a) Typical R_cell_-I_pulse_ curve obtained by varying the current pulse amplitude for a mushroom phase-change memory cell. Reprinted with permission from [174]. (b) Schematic illustrating the basic iterative programming concept using a sequence of adaptive write and verify steps. Reprinted with permission from [179].

These emerging advanced techniques have indeed triggered the recent progress of PRAM technology particularly in terms of its scalability. However, in order to outpace the growth rate of the current digital data, and to outperform other secondary storage devices, the available areal density of PRAM needs to be enhanced sharply, giving rise to the demand for the presence of more innovative technologies. At present, multi-level cell (MLC) storage and multi-layer 3D stacking are considered as two potential ways to realize the targets. Note that the resistance of PRAM in ‘SET’ state is mainly determined by the geometry and material properties of the electrodes, whereas its resistance in ‘RESET’ state is dominated by the size of the amorphized volume. As a result, different ‘RESET’ states with different amorphous volumes can be readily discriminated by measuring the cell resistance. This merit results in the invention of MLC in which the vast resistance contrast between the crystalline and amorphous phases would potentially allow for the formation of several intermediate resistance states appearing in one PRAM cell [174–176]. In this case, multiple bits can be stored in one cell, significantly reducing the cost per bit. The concept of a two-bit MLC is schematically interpreted in Figure 24. The logical states ‘0,0’ and ‘1,1’ corresponding to a complete ‘SET’ state (i.e. a fully crystallized phase-change layer) and a complete ‘RESET’ state (i.e. a fully amorphized phase-change layer), respectively. In between, there are two hybrid states, with amorphous volumes of different sizes, representing the logical states ‘0,1’ and ‘1,0’. The intermediate resistance level of the PRAM cell can be obtained from adjusting the writing pulse amplitude or the width of the trailing edge of a pulse, as shown in Figure 25(a). The insidious issue of this approach arises from the intrinsic device-to-device variations that mainly include inter-cell variations resulting from distributions in the thickness and lateral dimensions for different devices and intra-cell variations resulting from differences in both the atomic configuration of the amorphous regions and the distribution of polycrystalline nuclei and grain boundaries [177,178]. As a consequence, applying identical pulses to PRAM devices but with process and material variations would cause a widespread distribution of resistance levels across large PRAM arrays. One way to avoid this issue is to utilize an iterative programming scheme in which a write-and-verify sequence uses a feedback loop to minimize the error between the programmed and a specified target resistance, as revealed in Figure 25(b) [179]. The capability of achieving 16 intermediate resistance levels that stands for a 4-bit cell by means of MLC storage has already been demonstrated [180].

**Figure 26.**
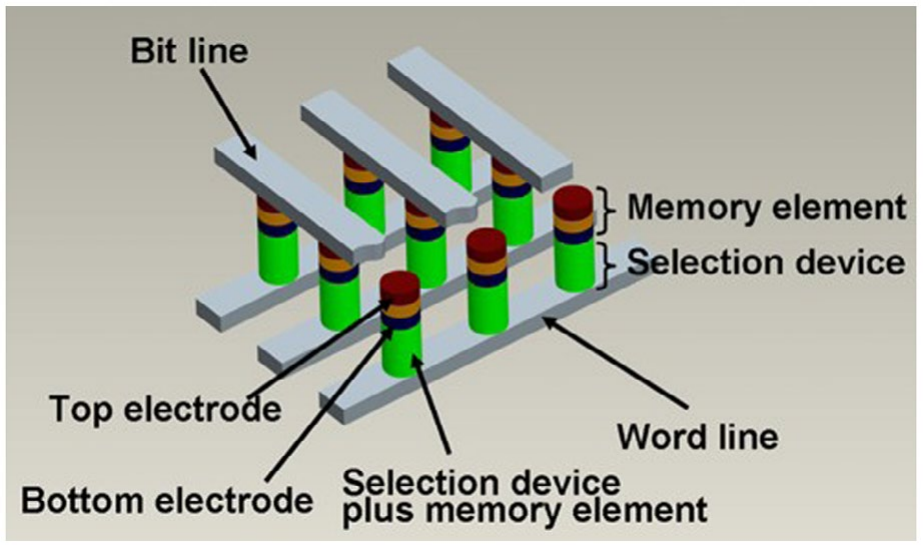
Structure of the cross-point PRAM cell. Reprinted with permission from [162].

**Figure 27.**
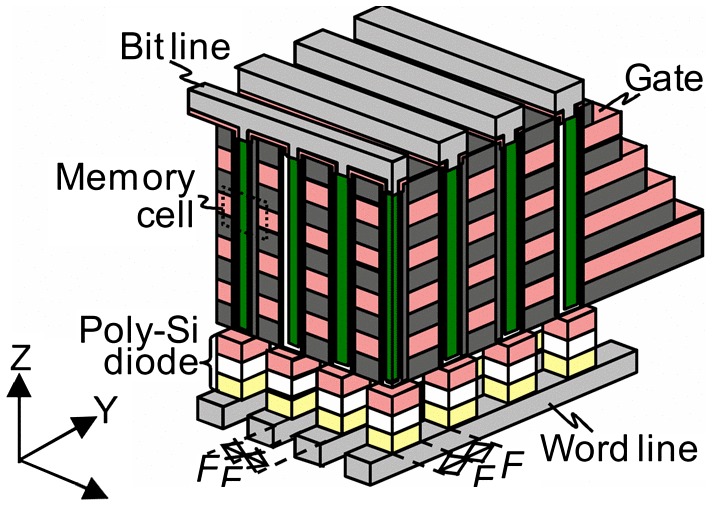
Device structure and operation of VCCPCM. Reprinted with permission from [190].

The pioneer of the 3D stackable phase-change memory with multiple stackable layers above each other was commonly considered to be the cross-point memory architecture comprising a phase-change layer sandwiched by a top electrode and a bottom electrode, integrated with a cell selector device connected to a row metal (word line) [181,182], as shown in Figure 26. The fact that the cell selector plays an important role in determining device density as well as the device programming current imposes several stringent requirements on the cell selector such as small contact area, infinitely off-state resistance, and high on-state conductivity. It is obvious that conventional MOSFET fail to provide the required current density with an average of 40 MA/μm^2^ for programming, and several alternative cell selectors such as bipolar transistors [181], p-n junction diodes [181], Schottky diodes [183], metal-insulator-transition [184], and ovonic threshold switching (OTS) [185] have previously undergone intensive study for the minimization of the memory selector while sustaining sufficiently high current density for programming. More recently one attractive access device, namely the mixed ionic-electronic conduction (MIEC) access device [186–188], has been proposed as a candidate for the 3D-stackable access device. MIEC-based access devices that make use of Cu ion motion in novel Cu containing MIEC materials can be fabricated with temperatures commensurate with back-end-of-line processing (~400°C), and their scalability to < 30 nm diameter and ability to conduct current density up to 50 MA/μm^2^ has also been demonstrated [189], rending them suitable for stacking of multi-layer PRAM arrays. Encouraged by these innovative technologies, a true 3D vertical chain cell type phase-change memory (VCCPCM) with the use of poly-Si diode, shown in Figure 27, has been developed [190]. The excellent scalability of the new PCMs used in VCCPCM while retaining a reasonable crystallization temperature makes it possible to reduce the cell size beyond the scaling limit of flash memory.

## 5. Main memory using PCMs

**Figure 28.**
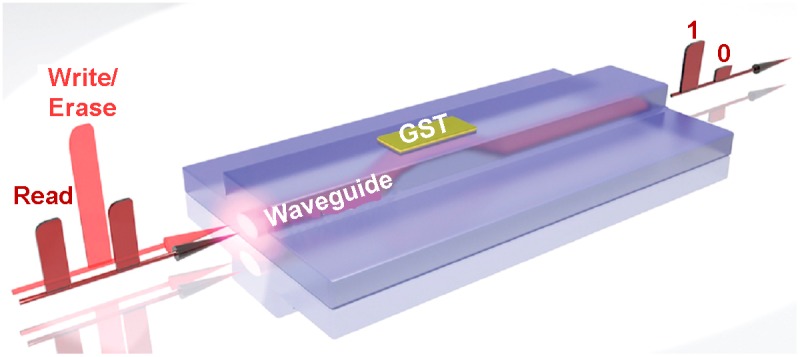
Schematics of the nanophotonic device using PCMs. Reprinted with permission from [11].

Light has been predominantly utilized for conveying information owing to its easy transmission in free space and exemption of crosstalk effect [191], which commercializes the use of optics for communication among multicore technology for the computer industry to overcome the bandwidth limitations of silicon electronics as well as power dissipation through electrical interconnects [192]. However, as the main memory is currently governed by the electronic storage mechanism, extra devices for light-to-electronics and electronics-to-light conversions are required for electronic computers, which would make the system cumbersome [11]. Therefore, the necessity to avoid optoelectronic conversions while reducing the latencies associated with electronic memories results in an imperative requirement for a true all-photonic memory that stores bits using photons rather than electrons. To achieve this goal, the pioneering concept of a non-volatile nanophotonic memory using PCMs has been theoretically proposed and experimentally verified [11], as illustrated in Figure 28. The device consists of a nanoscale phase-change layer (Ge_2_Sb_2_Te_5_ in this case) that is capped with a thin layer of indium tin oxide (ITO) to prevent oxidation, deposited on top of a half-etched waveguide on Si_3_N_4_ substrate optimized for single mode operation. The physics behind this novel nanophotonic memory device rely fully on the pronounced change in refractive index between the amorphous and crystalline states for different wavelengths [193]. According to this, the writing is performed by propagating a more intense light pulse along the waveguide whose energy can be partly absorbed by the Ge_2_Sb_2_Te_5_ element due to the evanescent coupling effect. The correlated phase transitions either from crystalline to amorphous phases or vice versa can therefore be achieved once the energy absorbed by Ge_2_Sb_2_Te_5_ is high enough to drive it towards transition temperature. Due to the refractive index variations between the two phases, the crystalline state usually presents higher attenuation and thus less optical transmission than the amorphous state [194]. Accordingly, this hybrid device will become more absorptive after crystallization, and give rise to a dramatic attenuation of the subsequent passing light pulse, while after amorphization the device is relatively less absorptive. Thanks to this, the stored data can be readily discriminated by measuring the transmission coefficient of the hybrid device by means of a low power optical pulse.

**Figure 29.**
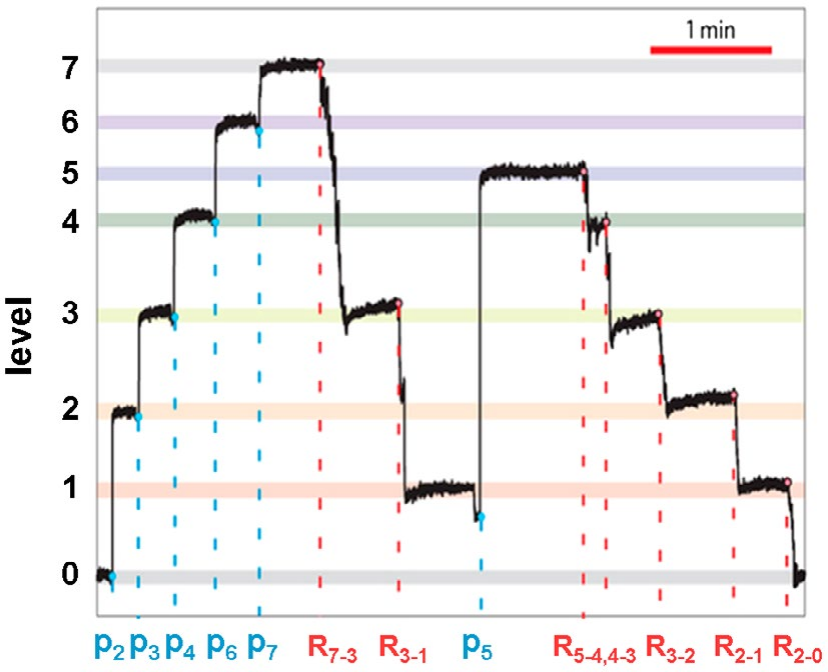
Eight clearly distinguishable levels are reached using this nanophotonic device by gradually changing the device transmission. Reprinted with permission from [11].

Unlike the pre-existing optical disc operated in the far field, the proposed nanophotonic phase-change memory is built in the near field and therefore not restricted in size by the diffraction limits of the applied optics pulse, enabling the scalability down to nanoscale dimension. Additionally, implementing the wavelength division multiplexed access (WDM) technique allows large arrays of all-optical memory elements to be integrated on nanoscaled on-chip waveguides [195,196], thus offering a prospective scheme towards ultra-fast all-optical data storage with ultra-low power consumption. The capability of providing fast switching speeds (500 ps), low power (480 fJ), single shot readout of the memory state, as well as repeated (×100) write/erase cycling while retaining high readout contrast for this hybrid phase-change photonic framework has been demonstrated [194]. More importantly, the potential of having multi-level access on this newly designed optical cell has also been tested, implying a high density storage function [194]. Obviously, the number of possible achievable levels is determined by the separation between the highest and lowest transmission states. In this case, according to the careful control of the accessible pulse energy, up to 8 levels physically indicated by different partial crystalline states that exhibit various transmission coefficients for a single optical cell were obtained, giving rise to Figure 29. It is essential to point out that the contrast or total change in transmission depends on the length of Ge_2_Sb_2_Te_5_, with the optimum length (along the waveguide) usually between 1 and 5 μm for high SNR in levels readout and low energy consumption [197].

## 6. Conclusions

As the pioneering phase-change product for storage applications, phase-change optical discs used to be representative of phase-change memories and received the pervasive applications predominantly in the fields of healthcare, media and entertainment, and education, due to its great portability and ability to store a large amount of data for a long period. Also, the inherent phase transition trait makes phase-change optical disc rewritable, thereby further strengthening its popularity from consumers’ perspectives. Although the drawbacks such as relatively low capacity and long data access time have ruled out the possible use of phase-change optical discs from main memory and secondary storage memory, it predominated the tertiary memory market during the last two decades when the total amount of archival data was much less than today. However, the advent of the age of ‘big data’ inevitably leads to a drastic increase in the amount of archival data that cannot be accommodated by the current phase-change optical disc technology due to the well-known diffraction limit in the far field. In this case, several advanced technologies such as near-field recording, multi-layer/side recording, and multi-level recording have been proposed to push forward the technology of phase-change optical discs and to extend their life in the data storage market. These technologies have improved the storage capacity of phase-change optical discs and made them viable for storage applications. In spite of these advancements, the state-of-the-art capacity of the phase-change optical disc is fixed at 60 GB for a double-sided disc and at 512 GB for a multi-layer disc [198], far behind the archival requirement. In addition to this, the gradual popularity of on-line cloud storage has further shrunk the existing demand for phase-change optical discs particularly for multi-media storage. The most desperate fact is that researchers seem to have lost interest in improving optical disc storage technology, as no comprehensive review on phase-change optical discs has been published since 2010. This fact may imply that the potential of phase-change optical discs for higher capacity has faded away and that the demise of the phase-change optical disc from the storage market is approaching.

As phase-change optical discs are gradually being withdrawn from the tertiary memory market, it is timely to develop a new memory device to fill such a gap and thus to outpace the storage requirement for the global archival market, triggering the invention of SPPCM that stores the data through phase transition induced by passing a current across a nanoscaled conductive tip. The use of a nanoscaled tip would confine the resulted mark into a region with a similar size to the tip and thus lead to an ultra-high capacity. Besides, SPPCM perfectly inherits the advantageous storage attributes of PCMs, enabling SPPCM to have fast switching speeds, low power consumption, a great endurance cycle, and long retention time. Because of these attractive features, SPPCM has been undergoing intensive research from both experimental and theoretical points of view, thereby allowing for a continual performance boost in both media stacks and probe tips. The feasibility of using SPPCM to achieve 3.8 Tb/in^2^ at laboratory level [132,199] and 10 Tb/in^2^ at simulation level [130] has been demonstrated respectively. The power consumption that was found to be < 100 pJ per bit is lower than other technologies, while the write speed for a single probe was expected to be 50 Mb/s with the assistance of a spinning disc for positioning medium [17], which can be significantly boosted by employing a probe array allowing multiple probes to write and read simultaneously. Despite these advantages, the concept of an SPPCM device still remains at the research level, the exact commercialized date for SPPCM is still hard to forecast, mainly due to the lack of deep understanding and thorough theoretical analysis of the role of the media stack and probe tip on the write/read operation of SPPCM. In addition to this, the relatively large cost of the initial system for SPPCM is another issue that needs to be treated seriously.

Today PRAM has successfully replaced phase-change optical discs to become the most famous phase-change memory and numerous publications and patents regarding PRAM technology have been delivered every year. The most stunning property of PRAM lies in its ability to retain all the aforementioned superior characteristics of PCMs at highly scaled dimension (several nm in thin-film thickness or nanoparticle diameter). Thanks to this, the scalability of PRAM down to < 5 nm in association with a switching speed of < 100 ns, and a cycle endurance of > 10^12^ at the cost of pico-scaled power consumption has been demonstrated [200,201]. Benefiting from the long-term efforts of worldwide researchers, the physical performances of PRAM have been subjected to continuous improvement, although it is still far behind its ultimate limits. Recently emerging technologies such as 3D stackable architecture and MLC recording have significantly compensated the adverse influence of the selector device on the resulting cell scalability, leading to further improvements in device programming, device read, cell design, and coding and signal processing approaches that can ensure the reliable retrieval of stored data. In spite of this encouraging progress, its ultimate target for dominating the secondary storage memory market is yet to be realized due to the simultaneous performance improvements of the other rival memory devices like HDDs [202]. The current consensus is that the state-of-the-art PRAM technology can readily satisfy the standalone flash memory requirement, and is even suitable for storage class memory (SCM), while its application for secondary storage memory is still very challenging in the presence of more revolutionary storage schemes.

The current main memory market is predominantly occupied by DRAM owing to its fast write time of <10 ns and superb endurance of >3 × 10^16^ cycles [203]. Under these circumstances it is not expected that electronic memories can outperform DRAM in the near future. Therefore, the necessity of developing a unique non-electronic memory to replace DRAM becomes apparent. Considering the ubiquity of fibre-optics networks and recent enthusiasm for optical computing, a concept of a novel on-chip nanophotonic memory using phase-change material has been proposed and fabricated experimentally. This hybrid device allows phase-change materials to be reliably switched between amorphous and crystalline states on an on-chip photonic waveguide, thus rendering it with some attractive features previously found on PRAM devices (e.g. great scalability, fast switching speeds, and low power consumption. Besides these well-known merits, the most exciting characteristics of this nanophotonic device arise from its pure optical operation governing the write/read functions, which therefore paves the way towards the main memory application for the future optical computer. Nevertheless, even if MLC technology has been introduced to this newly developed device, its achievable storage capacity still remains low, and may hamper its bright prospects.

## Disclosure statement

No potential conflict of interest was reported by the authors.
